# Social Structure and Interactions Differentially Shape Aerotolerant and Anaerobic Gut Microbiomes in a Cooperative Breeding Species

**DOI:** 10.1111/mec.70304

**Published:** 2026-04-10

**Authors:** Chuen Zhang Lee, Sarah F. Worsley, Terry Burke, Jan Komdeur, Falk Hildebrand, Hannah L. Dugdale, David S. Richardson

**Affiliations:** ^1^ School of Biological Sciences University of East Anglia Norfolk UK; ^2^ Centre for Microbial Interactions, Norwich Research Park Norwich UK; ^3^ Ecology and Evolutionary Biology, School of Biosciences University of Sheffield Sheffield UK; ^4^ Groningen Institute for Evolutionary Life Sciences (GELIFES) University of Groningen Groningen the Netherlands; ^5^ Quadram Institute Biosciences, Norwich Research Park Norfolk UK; ^6^ Earlham Institute, Norwich Research Park Norwich UK; ^7^ Nature Seychelles, Roche Caiman Mahé Seychelles

**Keywords:** *Acrocephalus sechellensis*, cooperative breeding, gut microbiome, social transmission, wild population

## Abstract

Social transmission of microbes has profound impacts on disease epidemiology and host health. However, how social factors influence gut microbiome (GM) transmission in wild populations is not well understood. Here, we use a wild population of the Seychelles warbler, a facultative cooperatively breeding passerine, to determine whether cooperative breeding behaviour influences the GM. Specifically, we hypothesise that close social interactions as part of cooperative breeding should encourage the sharing of anaerobic microbes, which may be less likely to transmit indirectly through the environment. We found that GM composition was more similar within versus between social groups, and this effect was driven by sharing both aerotolerant and anaerobic bacterial genera. As predicted, the similarity of anaerobic, but not aerotolerant, GM communities between pairs of individuals within a group was positively correlated with the strength of their social interactions (defined by their cooperative breeding status). Specifically, anaerobic GM composition was more similar between pairs of individuals that cooperate at the nest (dominant breeders and dominant‐helper pairs) than for non‐cooperative pairs (involving non‐helping subordinate individuals). This is likely because breeders and helpers directly interact while caring for offspring at a nest. This work reveals how cooperative social interactions lead to microbial transmission and thus contribute to shaping specific components of a host's gut microbiome.

## 1 Introduction

The vertebrate gut microbiota (GM) – the ecosystem of microbes that live within the gastrointestinal tract– plays a role in many important processes within the host, including metabolism, immune defences and cognition (Corbin et al. 2023; Zheng et al. 2020; Foster and McVey Neufeld 2013; Davies et al. 2022). In turn, many factors, such as host genetics, environment and diet, are important in shaping the GM (Davies et al. 2022; Bonder et al. 2016; Hicks et al. 2018; Grieneisen et al. 2021). Consequently, the GM can vary significantly not just across species and populations but also across individuals within populations (Hicks et al. 2018). Individual variation in GM composition has been associated with host health, being linked to, for example, nutrient extraction and immune function in vertebrates and, therefore, survival and reproductive success in wild animals (Zheng et al. 2020; Worsley et al. 2021; Cholewińska et al. 2020).

Despite evidence of the GM's significant role in host health and fitness (de Vos et al. 2022; Gould et al. 2018), there are still substantial gaps in our understanding of the factors that shape individual variation in GM composition. Among the least understood, yet potentially most important, factors is host sociality. The microbial metacommunity within social networks of hosts (the social microbiome) needs to be investigated to understand how social microbial transmission impacts host health and disease (Sarkar et al. 2024). To date, most research on microbial transmission across social networks has focused on pathogens, neglecting commensal microbes (Sarkar et al. 2020). In most vertebrates, the GM is initially acquired through parental transmission and then quickly becomes shaped by a combination of direct (via physical contact) and indirect (via the environment) transmission (see Sarkar et al. 2024). However, it is often difficult to distinguish between these mechanisms as socially interacting individuals also normally share the same environment (Raulo et al. 2024).

In captivity, conspecifics that socially interact share a more similar GM composition than those that do not (Hufeldt et al. 2010; Hildebrand et al. 2013; Bensch et al. 2023). However, captive animals are exposed to much less microbial diversity than their wild counterparts, which likely contributes to greater microbial sharing. Consequently, the GM of captive animals may be simpler (lower diversity and variation) than in nature and show many artefacts (Bensch et al. 2023). In contrast, wild animals encounter a much broader range of microbes due to factors such as exposure to other species, diverse and variable food sources, habitat and climatic variation and anthropogenic influences (Bensch et al. 2023; White et al. 2023). Very few studies have investigated the role of sociality in shaping the GM of wild animals, but see (Raulo et al. 2024, 2018; Archie and Tung 2015). Most work has focused on differences in GM between social groups (Raulo et al. 2018; Antwis et al. 2018; Bennett et al. 2016; Theis et al. 2012; Tung et al. 2015), but now we need to understand the links between GM and the degree of sociality within highly social animals.

Social organisation has also been associated with the microbiome communities of social insects (Gamboa et al. 2025; Shimoji et al. 2021; Jones et al. 2018) and non‐group‐living mice (Raulo et al. 2024, 2021), with individuals that interact more frequently having more similar microbial communities. Socially acquired GM similarity is likely driven by having a shared environment (indirect) and repeated social interactions (direct), such as grooming, food sharing and close contact (including copulations), which facilitate microbial transmission (Raulo et al. 2024, 2018; Dill‐McFarland et al. 2019). Related individuals that are from the same social group also have a more similar GM composition than unrelated individuals, highlighting the importance of host genetics in shaping the microbiome in groups (Grieneisen et al. 2021; Turnbaugh et al. 2009; Roche et al. 2023).

Aerotolerance may play a significant role in determining the likelihood of environmental versus direct transfer of microbial species (Raulo et al. 2024). Aerotolerant (aerobic and facultatively anaerobic) bacteria may grow outside the host and are therefore more likely to survive long enough to undergo indirect environmental social transmission (Mazel et al. 2024). By contrast, anaerobic bacteria survive less well outside the body and are likely limited to vertical and close‐contact transmission (Mazel et al. 2024; Moeller et al. 2018). Consistent with this, a couple of studies have suggested that social proximity facilitates the transfer of anaerobic bacteria (Raulo et al. 2024; Dill‐McFarland et al. 2019).

Some group‐living vertebrates practice cooperative breeding, whereby additional adult group members provide care to offspring produced by a limited number of breeders (often just a dominant pair) (García‐Ruiz et al. 2022; Cockburn 1998; Koenig and Dickinson 2016). Such subordinate ‘helpers’ enable dominant breeders to increase their reproductive success, while potentially providing the helpers with inclusive fitness benefits (including indirect [kin‐selected] and direct benefits, e.g., Cockburn 1998; Koenig and Dickinson 2016; Richardson et al. 2002). These ‘helpers’ interact closely with the breeders, potentially facilitating the direct transmission of microbes (Sarkar et al. 2024). However, given that helpers normally share the same space/territory and may be genetically related to the dominants (Cockburn 1998), separating the role of direct and indirect transmission in shaping the GM can be difficult. Research using suitable cooperative systems which allow these routes of transmission to be untangled and better understood is now needed.

Here, we use the facultatively cooperative breeding Seychelles warbler ( *Acrocephalus sechellensis* ) to assess how cooperative interactions shape individual GM variation. This system enables us to disentangle the effects of genetic relatedness from social interactions, as subordinates vary extensively in how related they are to the dominant breeders due to the frequent dispersal of offspring into non‐natal groups to become subordinates (Groenewoud et al. 2018), and even subordinates within their natal group being the result of extra‐pair paternity (Hadfield et al. 2006) and/or cobreeding (Raj Pant et al. 2019). In addition, as warblers are tree‐foraging insectivores, they are rarely exposed to other conspecifics' faeces, thus limiting non‐contact horizontal transfer post‐fledging. The insects they eat typically contain a high proportion of aerotolerant bacteria (Yun et al. 2014; Engel and Moran 2013). Therefore, we hypothesise that warblers will share aerotolerant bacteria through a shared environment, whereas close physical contact is needed to transfer anaerobic bacteria. We test the following predictions: (1) Individuals sharing a territory have more similar GM than those who do not. (2) Individual GM similarity is correlated with the closeness of the social relationship within the cooperative breeding system. (3) The cooperative relationship between individuals will more strongly affect the anaerobic, rather than the aerotolerant, GM components.

## 2 Materials and Methods

### 2.1 Study System

The Seychelles warbler population on Cousin Island (29 ha; 04° 20′ S, 55° 40′ E) has consisted of ca. 320 individuals from ca. 115 territories since 1985 (Brouwer et al. 2009; Kingma et al. 2016). This population has been extensively monitored during the minor (January–March) and major (June–October) breeding season each year, with the major season accounting for 94% of breeding (Brown et al. 2022; Komdeur 1992; Hammers et al. 2015). Since 1997, nearly all individuals (> 96%) have been uniquely marked with a combination of three colour rings and a British Trust for Ornithology metal ring (Hammers et al. 2015; Davies et al. 2021). The age of individuals is determined during their first catch, either directly when accessing them in the nest, or as begging fledglings, or using their eye colour (grey in chicks and fledglings [< 5 months], light brown in subadults [5–12 months] and red brown in adults [> 12 months]) (Komdeur 1992). Individuals almost never disperse between islands (Komdeur et al. 2004), and the annual resighting rate is high (98% ± 1% SE) (Raj Pant et al. 2020; Richardson et al. 2001).

Seychelles warblers often breed successfully in socially monogamous pairs (Komdeur 1996). Individuals who attain a breeding position typically remain in the same territory, defending it with the same partner until their death (Richardson et al. 2007). However, due to a shortage of suitable breeding opportunities, some individuals delay independent breeding and become subordinates, often, but not always, in their natal territory (Groenewoud et al. 2018; Komdeur 1992). In any given breeding event, some subordinates (20% males and 42% females; Hammers et al. 2019) contribute to alloparental care (defined as ‘helpers’), assisting with incubation (only females) and provisioning (both sexes), while others do not (non‐helper subordinates) (Komdeur 1992). Helpers benefit by gaining breeding experience through indirect fitness benefits (kin‐selected). Each season, every group member is given a breeding status: dominant male, dominant female, helper, non‐helper subordinate. Breeding attempts normally produce single egg clutches (80%) (Richardson et al. 2001). Extra‐group paternity occurs frequently (~44%) (Hadfield et al. 2006; Richardson et al. 2001). Fledglings leave the nest after 18–20 days but are provided with extended post‐fledgling care for up to 3 months (Richardson et al. 2001; Komdeur 1996; Komdeur et al. 2016).

Genetic relatedness of individuals within a group varies considerably (mean 0.26 ± 0.23 SD, range 0.00–0.77) because (a) not all subordinates are from the natal territory (Komdeur 1992), (b) subordinates hatched in the territory may be the result of extra‐pair paternity (Hadfield et al. 2006; Richardson et al. 2001) or subordinate maternity (Richardson et al. 2002, 2001; Raj Pant et al. 2019) and (c) dominant breeders are replaced over time when individuals die or are deposed (Richardson et al. 2007).

### 2.2 Sample Collection

Faecal samples were collected from 2017 to 2022 across 10 breeding seasons (Worsley, Davies, et al. 2024). Birds were captured in mist nets and placed in a clean disposable flat‐bottom paper bag containing a sterile metal grate covering a sterile plastic tray. This established protocol (Davies et al. 2022; Knutie and Gotanda 2018) allows any faecal sample that is produced by the bird to fall onto the plastic tray, minimising contact with the outside of the bird and the bag. After defaecation (ca. 15 min), the bird was released, and the sample was collected using a sterile flocked swab and placed in 1 mL of absolute ethanol in a sterile screw‐cap microcentrifuge tube. Control microbiome samples were taken from each fieldworker's hands by swabbing with a sterile flocked swab. Samples were stored at 4°C during the field season and transferred to −80°C for long‐term storage on reaching UEA. The time‐of‐day of each sample was recorded (minutes after sunrise—06.00 h GMT + 4), and the number of days between sampling and −80°C storage was recorded. A blood sample (ca. 25 μL) was collected from individuals during the same catch event (just before or after faecal sample collection) through brachial venipuncture and stored in 1 mL of absolute ethanol at 4°C.

### 2.3 Molecular Methods

Total genomic DNA was extracted from faecal samples using the Qiagen DNeasy PowerSoil Kit with a modified version of the manufacturer's protocol (see Davies et al. 2022). To minimise batch effects of extraction, samples were randomised. DNA was submitted for 16S rRNA amplicon sequencing using the amplicon libraries of V4 primers 515F (5′TGCCAGCMGCCGCGGTAA3′) and 806R (5′GGACTACHVGGGTWTCTAAT3′) and sequenced across seven batches using 2 × 250 bp, paired‐end sequencing on an Illumina MiSeq Platform (see detailed methodology in Davies et al. 2022; Worsley, Davies, et al. 2024). Control samples were also extracted and sequenced this way (*n* = 21 hand controls, 15 negative controls and 10 positive, ZymoBIOMICS Microbial Community Standard [D6300], controls).

DNA had previously been extracted from blood with the DNeasy blood and tissue kit (Qiagen) and used in molecular sexing (using the Chromo‐helicase‐DNA binding [CHD]‐W and CHD‐Z loci) (Sparks et al. 2022; Griffiths et al. 1998) and genotyping at 30 microsatellite loci for parentage analyses (Richardson et al. 2001; Sparks et al. 2022). All offspring hatched between 1991 and 2022 (2282 offspring, 1935 [85%] mothers, 2016 [88%] fathers) had been assigned parentage at > 80% confidence using *MasterBayes* 2.52 as part of previous studies (detailed in Hadfield et al. 2006; Sparks et al. 2022; Edwards et al. 2018). Relatedness between individuals was calculated from the *MasterBayes* pedigree using *sequoia* 2.11.4 in R Studio 2024.12.0 + 467 (Huisman 2017; R Core Team 2024; Posit team 2024).

### 2.4 Bioinformatics

The processing of DNA reads followed previously described steps using QIIME2 2019.10 (Worsley, Davies, et al. 2024; Bolyen et al. 2019). In brief, read truncation, filtering and classification into amplicon sequencing variants (ASV) was undertaken using DADA2 (Callahan et al. 2016). Taxonomic assignment of ASVs was performed using the naïve‐Bayes classifier on the SILVA 132 reference database (Quast et al. 2012). The resulting ASVs were imported to R using *phyloseq* 1.46.0 (Lahti and Shetty 2019; McMurdie and Holmes 2013). Samples were filtered to remove non‐bacterial sequences, reads not assigned to phylum level and potential contaminants (based on hand and lab controls). Potential contaminants that were detected in hand and negative controls were subsequently excluded from the faecal dataset using the prevalence‐based approach implemented in decontam v1.18.0 (Davis et al. 2018). Rarefaction curves (generated in iNEXT 3.0.1) demonstrated that samples reached > 95% sample completeness (whereby sequencing captured approximately 95% of ASV diversity) at 8000 reads (Worsley, Davies, et al. 2024). As such, 27 faecal samples with less than 8000 reads were removed and ASVs that had fewer than 50 reads across all samples were also removed, as these represented possible sequencing errors.

The aerotolerance status of each bacterial genus (1111 genera) was assigned using both Google Gemini 2.0 and ChatGPT 3.5 on 21 January 2025. Although aerotolerance may vary among species in some genera, aerotolerance is typically reported at the genus level in taxonomic references such as Bergey's Manual and in similar papers (Raulo et al. 2024; Mazel et al. 2024; Hanski et al. 2025). Accordingly, we assigned aerotolerance at the genus level. The text used was ‘Assign aerotolerance status for the following genera’, followed by the list of genera. Google Gemini returned a table of genera and aerotolerance statuses, while ChatGPT responded with text. ‘Facultative anaerobic’ and ‘Aerobic’ were categorised as ‘Aerotolerant’, ‘Anaerobic’ was categorised as ‘Anaerobic’ and everything else was categorised as ‘Unknowns’. After excluding unknown or unassigned genera (*n* = 891/1111 genera were successfully assigned), the accuracy of these assignments was checked by comparing the assignments obtained with the manually assigned genera in Raulo et al. (2024) using Bergey's Manual of Systematics of Archaea and Bacteria (Trujillo et al. 2015). The correspondence to the previous manual assignment in Raulo et al. (2024) using Google Gemini was 94.5% and ChatGPT was 74.2% (*n* = 145 or *n* = 98 genera, respectively). However, the assignments in (Raulo et al. 2024) could also have been incorrect or out of date. So, in addition, 80 random genera were manually checked using Bergey's Manual of Systematics of Archaea and Bacteria (Trujillo et al. 2015) by CL, and the correspondence was 96.3% for Google Gemini and 73.4% for ChatGPT. The assignments from Google Gemini were therefore used for subsequent analysis, please see the supplementary for the full list of assignments (Table S9).

### 2.5 Statistics

#### 2.5.1 GM Similarity Within and Between Breeding Groups

##### 2.5.1.1 Alpha Diversity

Both richness and Shannon diversity were calculated for each sample (after rarefying each sample to 8000 reads) at the ASV level using *phyloseq* 1.46.0 (McMurdie and Holmes 2013). A pairwise alpha diversity difference was calculated for ASV richness and Shannon diversity, which were made negative to reflect similarities of pairwise alpha diversity metrics. Importantly, samples were then filtered to include only sample pairs from individuals from the same field period (*n* = 27,821 pairwise comparisons across 648 samples from 345 birds) to control for temporal variation. There were 10 field periods, each defined by the season within a year (a major and minor breeding season occurs each year) and year of sample collection. Across these periods, we generated an average of 3091 ± 993 SE pairwise comparisons per field period. A linear mixed effect multi‐membership model (*lmer* with *lmerMultiMember*) using *lme4* 1.1‐35.5 (Bates et al. 2015) was used to test whether the difference in alpha diversity was smaller when pairs were from the same breeding group than between breeding groups; this may arise due to several factors including the social transmission of microbes. Breeding group status (within a group, between groups), the age difference of individuals (0–16.7 years), sex difference (no/yes), the number of days apart samples were collected (0–97 days), the difference in the time of day samples were collected (0–634 min), season (minor/major), present in nest at hatch (whether one individual was present in the other's nest at hatch, e.g., as a sibling, helper or parent), and relatedness were included as explanatory variables; summary of sample metadata is included in Table SM1. Sample year and a multi‐membership ID (generated by lmerMultiMember, combining the bird IDs from both birds in a dyad into a single random variable, to account for the repeated occurrences of birds in different dyads; van Paridon et al. 2023), were used as random variables. Hereafter, all models included the same explanatory and random variables unless stated otherwise. Variance inflation factor (VIF) scores were computed to test for collinearity among the terms (all VIF scores were < 3).

##### 2.5.1.2 GM Composition

Differences in GM composition (beta diversity) at the ASV level were modelled using the same pairwise approach as for alpha diversity. Unrarefied raw reads were filtered to remove rare taxa (< 5% occurrence), and then centered log ratio (CLR) transformed using *microbiome* 1.20.0, which controls for differences in library size and is suitable for compositional datasets (Gloor et al. 2017). A pairwise Aitchison distance matrix was then calculated using *phyloseq* 1.46.0 (Callahan et al. 2016; McMurdie and Holmes 2013), and made negative to reflect GM composition similarity. A multi‐membership lmer was used to test if samples from individuals within a group had more similar GM composition compared to those outside of the group, where GM Aitchison distance was used as a response variable and the explanatory and random variables were as described for alpha diversity above.

##### 2.5.1.3 Aerotolerance

Bacterial taxa were split into an anaerobic dataset (205 anaerobic genera) and an aerotolerant dataset (686 aerotolerant genera). The same model structure (between/within breeding group GM composition model) was used to determine if within‐group changes in GM composition were dependent on aerotolerance capability.

#### 2.5.2 The GM and Social Status Categories

##### 2.5.2.1 Alpha Diversity

A second alpha diversity model was constructed as above (between breeding group alpha diversity model) but replacing breeding group status with individual status. This model tested whether particular status groups had more similar pairwise alpha diversity values which could arise due to greater levels of social contact (e.g., between breeders and helpers). Pairs of samples were filtered from distance matrices to only include comparisons made within the same breeding group (*n* = 279 pairwise comparisons across 322 samples from 204 individual birds). There were five groupings for individual status pairs: (1) dominant breeding pair (Dom‐Dom), (2) breeders—helpers (Dom‐Help), (3) dominant breeders—other subordinates (Dom‐Sub), (4) helpers—other subordinates (Help‐Sub), (5) subordinates—subordinates (Sub‐Sub). A linear mixed‐effect multi‐membership model was used, with pairwise differences in alpha diversity as the response variable. Individual status, the age difference of individuals, sex difference, the number of days apart samples were collected, the difference in the time of day samples were collected, season, present in nest at hatch and relatedness were included as explanatory variables. Sample year and a multi‐membership ID were used as random variables. If the overall individual status pair predictor term was significant, a post hoc pairwise comparison was performed using a Tukey test.

##### 2.5.2.2 Overall GM Composition

A social status category model was constructed to assess the impact of individual status on GM composition by replacing breeding group status with individual status comparisons and restricting comparisons to within‐breeding group. A multi‐membership lmer was used, where GM Aitchison distance was used as a response variable. Exactly the same explanatory and random variables were used as for the social status alpha diversity model described above.

##### 2.5.2.3 Aerotolerance Versus Anaerobic GM Composition

The same model structure as directly above was used to test whether patterns of GM composition associated with within‐group social status categories differed according to bacterial aerotolerance capability. Finally, the same model was run but lumping the within group social status categories to compare all categories that involved the pair of individuals interacting at a shared nest (Dom‐Dom and Dom‐Help combined) with all pairs that did not (Dom‐Sub, Help‐Sub, Sub‐Sub combined), using the same model structure as above.

## 3 Results

### 3.1 GM Similarity Within Versus Between Breeding Groups

#### 3.1.1 Alpha Diversity

The pairwise differences in observed ASV richness and Shannon diversity did not significantly differ between pairs of individuals from within the same breeding group versus pairs from different breeding groups (Table S1, Table 1). The pairwise differences in ASV richness and Shannon diversity were negatively associated with the number of days between sampling points increased (Estimate = −0.001, *p* < 0.001, Table S1, Table 1). The pairwise differences in the Shannon diversity metric were also marginally associated with season (i.e., individual diversity was more similar in the minor than major season, Estimate = −0.065, *p* = 0.053) and time in season (negatively, Estimate < 0.001, *p* = 0.050) (Table 1).

**TABLE 1:** A linear mixed effect model (lmer) investigating the relationship between breeding group membership and gut microbiome ASV Shannon diversity similarity in pairs of Seychelles warblers (*N* = 27,821 pairwise comparisons across 648 samples from 345 individual birds).

Characteristic	Beta	SE	Statistic	df	*p*
**(Intercept)**	**−1.279**	**0.072**	**−17.7**	**12.5**	**< 0.001**
Breeding group (between/within)	−0.012	0.058	−0.206	27,548	0.837
Age difference	0.001	0.003	0.496	24,508	0.620
Sex (same/different)	−0.006	0.011	−0.567	27,560	0.571
*Season (major/minor)*	*−0.065*	*0.033*	*−1.94*	*1654*	*0.053*
*Time of day*	*< 0.001*	*< 0.001*	*−1.96*	*27,712*	*0.050*
**Time in season**	**−0.001**	**< 0.001**	**−3.98**	**27,775**	**< 0.001**
Relatedness	−0.029	0.087	−0.333	27,582	0.739
Shared nest at hatch (no/yes)	−0.010	0.025	−0.381	26,525	0.703

Random	27,821 observations		Variance
Multi membership ID	(Intercept)	345 groups	0.374
Sample year	(Intercept)	6 years	0.137
Residual			0.880

*Note:* Significant terms (*p* < 0.05) are in bold, marginal terms (*p* < 0.10) in italics. Reference categories for categorical variables were the first term in brackets. Time of day was measured as minutes apart, and time in season was measured as days apart.

#### 3.1.2 GM Composition

**FIGURE 1:**
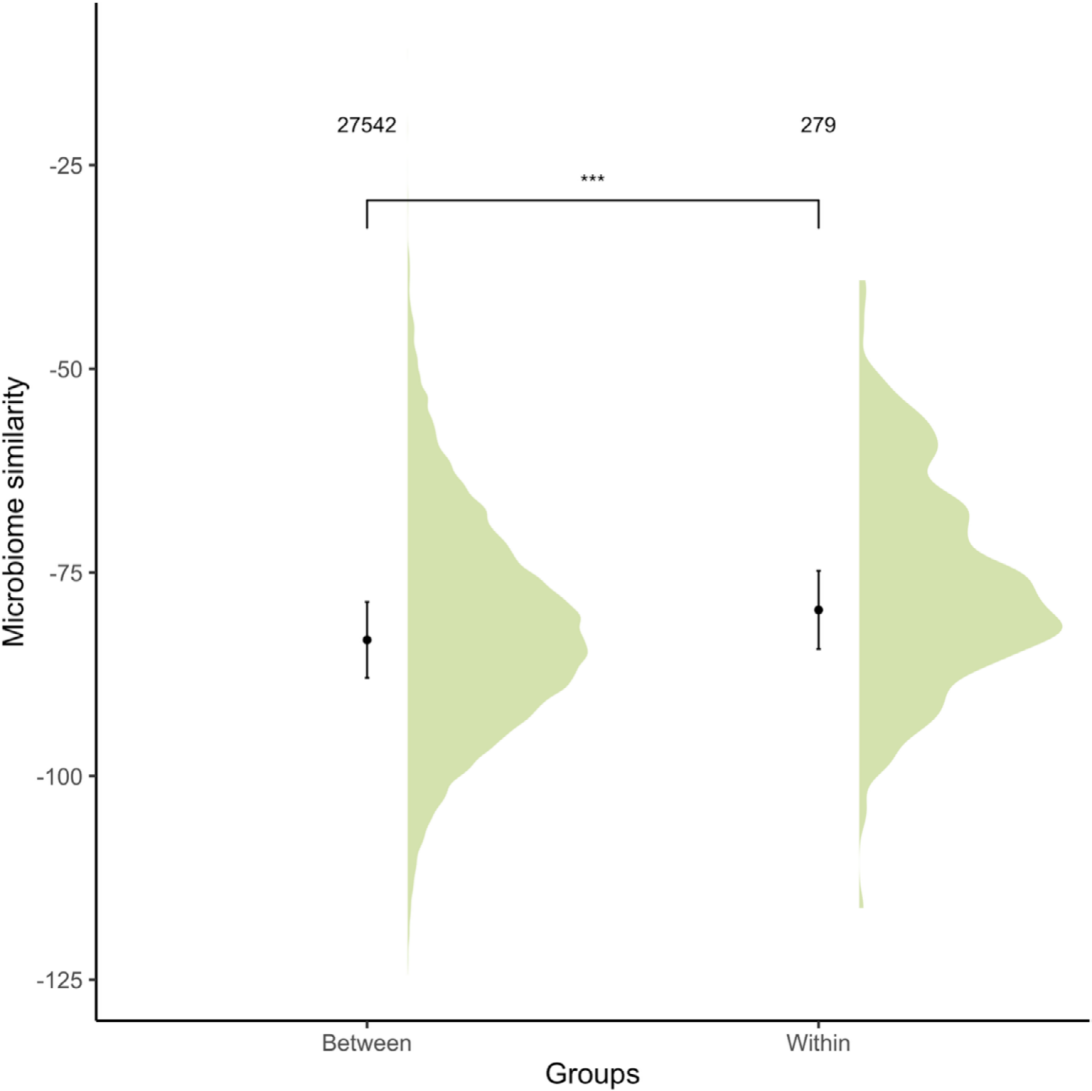
Gut microbiome composition similarity of pairs of individuals from the same versus pairs of individuals taken from different breeding groups in the Seychelles warbler (*N* = 27,821 pairwise comparisons across 683 samples from 345 individual birds). Dots and lines represent model predictions with 95% confidence intervals calculated from lmer models. The density plot represents the distribution of raw data. ***Represents *p* < 0.001 from model predictions (Table 2).

Pairs within breeding groups had a more similar GM composition (CLR‐transformed Atchison distance) than pairs in different breeding groups (Estimate = 3.68, *p* < 0.001, Table 2, Figure 1). Additionally, pairs sampled in the minor season had a more similar GM composition compared to pairs sampled in the major season (Estimate = 2.06, *p* < 0.001, Table 2). GM composition became increasingly different between individuals as the number of days between sampling of each of the pair increased (Estimate = −0.007, *p* < 0.038, Table 2). Moreover, individuals that shared a nest at hatch (including from different seasons; as either siblings, parents or helpers) had a significantly more similar GM composition (Estimate = 0.538, *p* < 0.036, Table 2).

**TABLE 2:** A linear mixed effect model investigating gut microbiome composition similarity in Seychelles warbler pairs from the same versus pairs from different breeding groups (*N* = 27,821 pairwise comparisons across 648 samples from 345 individual birds).

Characteristic	Beta	SE	Statistic	df	*p*
**(Intercept)**	**−83.21**	**2.38**	**−35.0**	**6.17**	**< 0.001**
**Breeding group pair (between/within)**	**3.683**	**0.581**	**6.34**	**27,490**	**< 0.001**
Age difference	0.016	0.028	0.556	27,767	0.578
Sex (same/different)	−0.123	0.109	−1.13	27,493	0.259
**Season (major/minor)**	**2.062**	**0.353**	**5.84**	**25,345**	**< 0.001**
Time of day	< 0.001	< 0.001	−0.304	27,572	0.761
**Time in season**	**−0.007**	**0.003**	**−2.08**	**27,590**	**0.038**
Relatedness	0.494	0.870	0.568	27,502	0.570
**Shared nest at hatch (no/yes)**	**0.538**	**0.257**	**2.09**	**27,806**	**0.036**

Random	27,821 observations		Variance
Multi membership ID	(Intercept)	345 groups	6.898
Sample year	(Intercept)	6 years	5.514
Residual			8.808

*Note:* Significant terms (*p* < 0.05) are in bold. Reference categories for categorical variables were the first term in the brackets. Time of day was measured as minutes apart, and time in season was measured as days apart.

#### 3.1.3 Aerotolerant Versus Anaerobic Bacteria

Considering aerotolerant bacterial genera, GM compositional similarity was significantly higher in pairs from the same breeding group compared to pairs from different breeding groups (Estimate = 1.96, *p* < 0.001, Table 3). Aerotolerant GM composition was also significantly less similar with increasing age differences (Estimate = −0.098, *p* < 0.001), time of day difference (Estimate < 0.001, *p* = 0.033) and time in season difference (Estimate = −0.006, *p* = 0.001), but more similar if the pair shared a nest at hatch (Estimate = 0.312, *p* = 0.031, Table 3).

**TABLE 3:** A linear mixed effect model (lmer) investigating the relationship between aerotolerant gut microbiome composition similarity in pairs of Seychelles warblers from the same breeding group versus pairs generated from individuals sampled from different breeding groups (*N* = 27,821 pairwise comparisons across 648 samples from 345 individual birds).

	Estimate	SE	df	*t*	*p*
**(Intercept)**	**−46.49**	**1.10**	**7.04**	**−42.4**	**< 0.001**
**Breeding group pair (between/within)**	**1.957**	**0.325**	**27,489**	**6.02**	**< 0.001**
**Age difference**	**−0.098**	**0.007**	**27,603**	**−13.3**	**< 0.001**
Sex (same/different)	−0.019	0.061	27,492	−0.317	0.752
Season (major/minor)	0.273	0.197	22,916	1.38	0.167
**Time of day**	**< 0.001**	**< 0.001**	**27,566**	**−2.13**	**0.033**
**Time in season**	**−0.006**	**0.002**	**27,583**	**−3.41**	**0.001**
Relatedness	0.756	0.486	27,498	1.55	0.120
**Shared nest at hatch (no/yes)**	**0.312**	**0.145**	**27,803**	**2.16**	**0.031**

Random	27,821 observations		Variance
Multi membership ID	(Intercept)	345 groups	16.018
Sample year	(Intercept)	6 years	6.029
Residual			24.243

*Note:* Significant terms (*p* < 0.05) are in bold. Reference categories for categorical variables were the first term in the bracket. Time of day was measured as minutes apart, and time in season was measured as days apart.

Considering only anaerobic bacterial genera, pairs within the same breeding group had more similar GM compositions compared to pairs from separate breeding groups (Estimate = 0.844, *p* = 0.003, Table 4). The anaerobic GM composition was significantly negatively associated with increasing time of day difference (Estimate = −0.001, *p* = 0.001), and time in season difference (Estimate = −0.007, *p* < 0.001) but was more similar if the pair shared a nest at hatch (Estimate = 0.266, *p* = 0.035, Table 4).

**TABLE 4:** A linear mixed effect model (lmer) investigating the relationship between anaerobic gut microbiome composition similarity in pairs of Seychelles warblers from the same breeding group versus pairs generated from individuals sampled in different breeding groups (*N* = 27,821 pairwise comparisons across 648 samples from 345 individual birds).

	Estimate	SE	df	*t*	*p*
**(Intercept)**	**−24.53**	**0.807**	**6.45**	**−30.4**	**< 0.001**
**Breeding group pair (between/within)**	**0.844**	**0.285**	**27,179**	**2.96**	**0.003**
Age difference	−0.002	0.006	27,370	−0.366	0.714
Sex (same/different)	0.061	0.053	27,185	1.14	0.255
Season (major/minor)	−0.247	0.170	19,017	−1.45	0.147
**Time of day**	**−0.001**	**0.000**	**27,310**	**−3.38**	**0.001**
**Time in season**	**−0.007**	**0.002**	**27,337**	**−4.34**	**< 0.001**
Relatedness	−0.431	0.425	27,196	−1.01	0.310
**Shared nest at hatch (no/yes)**	**0.266**	**0.126**	**27,326**	**2.11**	**0.035**

Random	27,821 observations		Variance
Multi membership ID	(Intercept)	345 groups	6.342
Sample year	(Intercept)	6 years	3.408
Residual			18.298

*Note:* Significant terms (*p* < 0.05) are indicated in bold. Reference categories for categorical variables were the first term in brackets. Time of day was measured as minutes apart, and time in season was measured as days apart.

### 3.2 The GM and Within‐Group Social Status Categories

#### 3.2.1 Alpha Diversity

**FIGURE 2:**
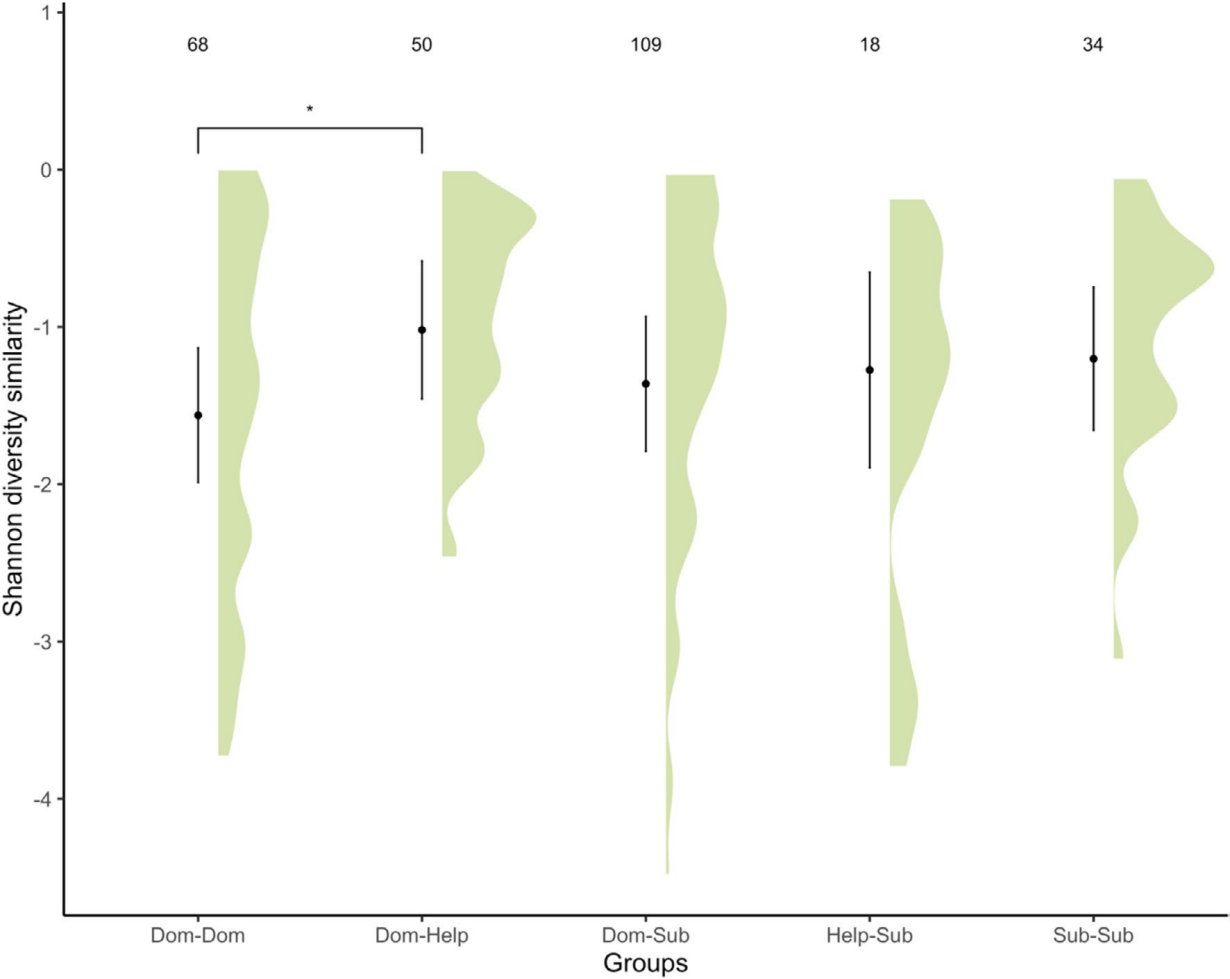
Gut microbiome Shannon diversity similarity of different breeding group status pairs of Seychelles warblers. Dots and lines represent model predictions with 95% confidence intervals calculated from lmer models. The density plot represents the distribution of raw data. *N* = 279 pairwise comparisons across 322 samples from 204 individual birds. *Represents *p* < 0.05 from model predictions (Table S3).

We assessed pairwise differences in ASV richness (Table S2) and Shannon diversity (Table S3) between pairs of birds with different statuses within the same breeding group. Only Shannon diversity was significantly more similar for dominant‐helper status pairs than for dominant pairs (Estimate = 0.542, *p* = 0.017, Table S3, Figure 2). All other pairwise comparisons were not significantly different from each other (Tables S2–S4) and lower than for dominant‐helper status pairs.

#### 3.2.2 Overall GM Composition

None of the social status pair categories significantly differed in overall GM composition similarity (Table S5).

#### 3.2.3 Aerotolerant Versus Anaerobic GM Composition

Pairwise similarities in aerotolerant GM composition did not differ between social status pair categories (Table S6). The only significant effect in this model was a negative association between aerotolerant GM composition similarity and increasing differences in host age (Estimate = −0.245, *p* < 0.001, Table S6).

**FIGURE 3:**
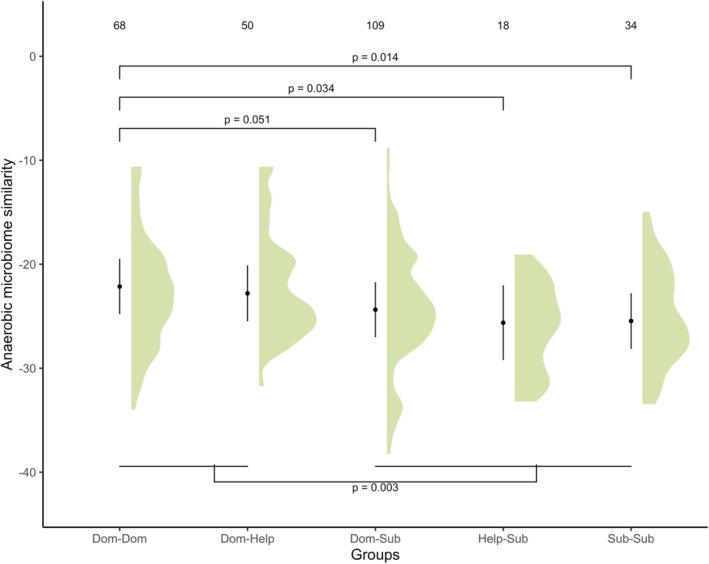
Anaerobic gut microbiome composition similarity of different social status pair categories of Seychelles warblers (comparison within groups). Dots and lines represent model predictions with 95% confidence intervals calculated from lmer models. The density plot represents the distribution of raw data. *N* = 279 pairwise comparisons across 322 samples from 204 individual birds. *p*‐values between categories shown above the plots (from model predictions; Table 5) and nest‐sharing groups of categories shown below the plots (Table S7) are shown with brackets.

In contrast, anaerobic GM composition similarity did significantly differ between social status pair categories (Table 5, Figure 3). Specifically, the anaerobic GM compositional similarity of dominant‐dominant and dominant‐helper categories did not differ (Table 5, Figure 3). However, anaerobic GM composition was significantly more similar in dominant‐dominant pairs than for pairs in the other three categories (dominant‐subordinate [marginal, Estimate = −2.231, *p* = 0.051], helper‐subordinate [Estimate = −3.483, *p* = 0.034] and subordinate‐subordinate pairs [Estimate = −3.319, *p* = 0.014]) (Table 5, Figure 3). The anaerobic GM composition was not significantly different in all other pairwise comparisons (Table S7).

**TABLE 5:** A linear mixed effect model (lmer) investigating the relationship between individual breeding group status pairs and anaerobic GM composition similarity of Seychelles warblers (*N* = 279 pairwise comparisons across 320 samples from 204 individual birds).

Characteristic	Beta	SE	Statistic	df	*p*
**(Intercept)**	**−22.44**	**1.30**	**−17.3**	**39.0**	**< 0.001**
Individual status pair					
Dom—Dom	—	—	—		
Dom—Help	−0.661	1.23	−0.539	209	0.590
*Dom—Sub*	*−2.231*	*1.14*	*−1.96*	*194*	*0.051*
**Help—Sub**	**−3.483**	**1.63**	**−2.13**	**160**	**0.034**
**Sub—Sub**	**−3.319**	**1.34**	**−2.47**	**189**	**0.014**
Age difference	0.009	0.067	0.135	258	0.893
Sex (same/different)	0.335	0.735	0.456	239	0.649
Season (major/minor)	0.049	1.05	0.046	91.8	0.963
Time of day	−0.002	0.003	−0.591	250	0.555
Time in season	0.001	0.018	0.083	260	0.934
Relatedness	1.622	1.82	0.893	194	0.373
Shared nest at hatch (no/yes)	−0.283	0.863	−0.328	233	0.743

Random	274 observations		Variance
Multi membership ID	(Intercept)	204 groups	1.836
Sample year	(Intercept)	6 years	1.576
Residual			4.341

*Note:* Significant terms (*p* < 0.05) are indicated in bold, marginal terms (*p* < 0.1) are indicated in italics. Reference categories for categorical variables were the first term in brackets. Time of day was measured as minutes apart, and time in season was measured as days apart.

Finally, when combining the nest‐sharing pairs and the non‐nest‐sharing pairs into two overall categories, anaerobic GM composition similarity was higher for nest‐sharing pairs (Dom‐Dom and Dom‐Help) than for non‐nest‐sharing pairs (Dom‐Sub, Help‐Sub, Sub‐Sub) (Estimate = −2.317, *p* = 0.003, Table S8).

## 4 Discussion

We investigated how sociality shapes the GM in the cooperative breeding Seychelles warbler. GM alpha diversity did not differ between individuals from the same breeding group or individuals from different breeding groups. However, individuals within a group had a more similar GM composition compared to individuals from different groups. When separating aerotolerant from anaerobic bacteria, individuals within a breeding group shared more of both categories than did individuals from different groups. When we focus on cooperative breeding status differences within breeding groups, dominants and helpers shared more similar pairwise measures of GM alpha diversity than the dominant pair, but no other pairs were significantly more similar in terms of their GM diversity. When looking at all GM genera, we found no differences in GM compositional similarity between any of the within‐group social status categories. However, when separating aerotolerant and anaerobic bacterial genera, we find that, as predicted, anaerobic GM composition was more similar between birds that directly cooperate during breeding and thus interact closely at the nest than between categories of pairs that interact less.

Seychelles warbler groups have defined territory boundaries that they defend year‐round to secure resources (Hammers et al. 2019). Levels of GM pairwise differences in alpha diversity metrics did not differ when comparing individuals sampled from the same or different groups. This suggests that factors associated with living in the same breed group (e.g., shared environment, genetic similarity and greater social transmission) do not influence the alpha diversity of an individual's GM. However, GM alpha diversity is just the number (richness) or relative abundance (Shannon) of GM ASVs within individuals, can be highly variable and may not reflect shared GM composition; indeed, individuals that live in different territories can have differing GM composition but still have the same alpha diversity and similarities in alpha diversity do not necessarily mean shared microbial taxa (Worsley, Lee, et al. 2024; Johnson and Burnet 2016). As such, an analysis of bacterial composition provides stronger evidence of the role of social transmission in shaping the GM.

As predicted, GM composition was more similar for individuals from the same groups than individuals from different groups, even when controlling for relatedness. Recent research on the social transmission of microbes in other group‐living animals has yielded similar results (Raulo et al. 2018; Tung et al. 2015). This increase in GM composition similarity within groups likely arises from such individuals sharing the same resources, but also because of increased physical interaction among individuals. Indeed, non‐group living wild mice ( *Apodemus sylvaticus* ) that interact more frequently tend to share a more similar microbiome composition (Raulo et al. 2024, 2021). In our results, that both aerotolerant and anaerobic bacterial communities were more similar within than between breeding groups further supports the idea that shared microbes occur because of a combination of shared environment/diet (e.g., aerobes from insects) and close physical contact (e.g., the transmission of anaerobes). However, it would be challenging to distinguish between resource sharing and social contact modes of transmission when only comparing between and within social groups, as the two modes would overlap (but see below).

Associations between GM characteristics and social interactions have been previously reported in social insects, the harvester ants (*Veromessor andrei*) and honey bees ( *Apis mellifera* ) (Gamboa et al. 2025; Jones et al. 2018), wild baboons ( *Papio cynocephalus* ) (Tung et al. 2015) and wild mice (Raulo et al. 2024), but researchers have not directly investigated social interactions within cooperative breeders. In social systems where cooperative breeding occurs, a hierarchy of closeness of interactions between individuals exists, with the dominant breeding pair interacting most frequently, followed by breeders‐helpers, breeders‐non‐helping subordinates, helpers‐non‐helping subordinates and subordinates‐subordinates (Cant and Field 2005; Komdeur 1994). Interestingly, in Seychelles warblers, breeders‐helpers have a more similar GM diversity than do the dominant breeding pair. However, similarities in GM diversity do not imply shared GM composition. This may be because the helpers (who are normally female) also share in incubating with the dominant female (Richardson et al. 2001) while male dominants do not. Importantly, when comparing all bacterial genera, GM compositional similarity was not associated with the closeness of cooperative breeding relationships within a group. This may be because individuals from the same environment tend to have a similar diet, which leads to homogenisation of the GM irrespective of social interactions. However, as predicted, if we only focus on anaerobic genera we do find that the closeness of cooperative breeding relationships influences GM composition similarity. This was not the case for the aerotolerant GM. These results support the hypothesis that aerotolerant microbes are likely transmitted through a shared general environment (i.e., the territory), while anaerobic microbes require closer social interactions, such as direct interactions at the nest, for transmission. The logic being that oxygen‐sensitive anaerobic bacteria do not survive long outside of a host and therefore require close direct contact for transmission (Raulo et al. 2024). Our findings concur with previous work that investigated anaerobic versus aerotolerant GM similarity in relation to social intimacy using GPS data tracking or grooming behaviour (Raulo et al. 2024; Tung et al. 2015).

Is there likely to be any benefit of GM transmission through close social interactions in cooperatively breeding species? One benefit may be gaining beneficial anaerobic microbes. Anaerobic gut microbes are more likely to form close symbiotic relationships with their host as they cannot survive in the aerotolerant conditions outside of the intestinal tract. Indeed, most probiotics—living microbes that provide health benefits—are anaerobic bacteria (El Enshasy et al. 2015). Benefits include aiding gut homeostasis and aiding digestion (Nalla et al. 2022; Kelsey and Colpoys 2018; Zhang et al. 2016) and supporting the host's immune system by preventing pathogens from colonising the GM (Murata et al. 2025; Wells et al. 1988). However, there are also potential downsides to increased transmission, such as pathogen transmission. Although many life‐threatening pathogens are aerotolerant (André et al. 2021), previous studies tracking pathogen transmission have suggested that there is an increased risk of spread in animals due to social proximity and shared resources (Duncan et al. 2021; Lebarbenchon et al. 2015). In the Seychelles warbler, ASVs that were found to be more abundant in birds that died in a previous study were all aerotolerant (*n* = 8 genera; *Microlunatus*, *Rubrobacter*, *Kocuria*, *Microbacterium*, *Mycobacterium*, *Methylobacterium*, *Aureimonas*, *Rubellimicrobium*) (Worsley et al. 2021). Therefore, it is unlikely that the increased transmission of anaerobic bacteria due to social interactions is a major factor contributing to mortality or pathogen transmission in this context.

The Seychelles warbler is an excellent system for studying the social transmission of the GM. However, several limitations exist, such as samples not always being collected from all individuals within a breeding group within the same field period. All tests were restricted to samples within the same field seasons to ensure that individuals had the opportunity to interact recently, and in a similar environment, as temporal effects are known to influence GM communities in the Seychelles warbler, as well as other wild animals (Hicks et al. 2018; Worsley, Davies, et al. 2024; Marsh et al. 2022). Furthermore, although the finding that social closeness makes anaerobic GM composition more similar is clear and important, incorporating shotgun metagenomic data would help determine whether differences in taxonomy alter GM function and the possible contribution of these microbes to host health (Worsley, Mazel, et al. 2024). Additionally, metagenomics would enable the analysis of the GM at the species or strain‐level (Anyansi et al. 2020), which would provide higher resolution when asking how GM components are correlated with social closeness rather than environmental transmission. Strain‐tracking between family members and how long strains persist in the GM during an individual life would also improve our understanding of how social closeness shapes the GM (Hildebrand et al. 2021). However, the overall patterns as detected in our study are still valid and shotgun metagenomics for the number of samples required would be very costly. In addition, the use of GPS logger data would allow us to generate more nuanced social networks and determine the strength of social relationships (Kingma et al. 2016). Unfortunately, GPS monitoring of Seychelles warblers within territories is not yet effective, as the accuracy of current tracking technology (i.e., sufficiently light weight to use on the birds) relative to the size of the Seychelles warbler's extremely small territories (0.18–0.46 ha per territory; Komdeur and Pels 2005), limits our ability to track individual interactions. Given the quality of the data on the Seychelles warblers gained through intense fieldwork observations, we are confident of the reliability of our estimates used here regarding the closeness of relationships between individuals (Brouwer et al. 2009; Hammers et al. 2019; Komdeur 1994).

Overall GM composition was also more similar between adult pairs when one individual (parent/helper) had previously (in a different season) attended the other as a nestling, suggesting that the developmental GM tends to persist and remains more similar in later life due to the earlier shared natal environment. This finding is consistent with that found in humans, where an individual shares gut microbial strains with their mothers and these are maintained throughout life (Valles‐Colomer et al. 2023; Eikenaar et al. 2007).

In the present study on the Seychelles warbler, when assessing the GM both within and across groups, relatedness was not a predictor of GM composition similarity. This may be because highly related individuals, such as siblings, may not share the same territory later in life when we sample them (all samples were post‐fledgling), especially since most individuals disperse from their natal territory as soon as a breeding opportunity elsewhere becomes available (Eikenaar et al. 2007). In wild mice and Verreaux's sifaka ( *Propithecus verreauxi* ), kinship and relatedness did not predict GM similarity (Raulo et al. 2021; Perofsky et al. 2017). However, in humans and wild baboons, related individuals share more similar GMs (Grieneisen et al. 2021; Turnbaugh et al. 2009; Roche et al. 2023).

The Seychelles warbler GM was also influenced by environmental variables, especially the number of days apart that samples were collected, which is consistent with previous studies on this species (Worsley, Davies, et al. 2024; Worsley, Lee, et al. 2024; Lee et al. 2025). The effect of this variable on GM diversity and composition could be explained by changes in weather and food availability throughout the season or the storage time of our samples (Cunningham et al. 2020). However, we cannot separate these two possibilities as they are strongly correlated. Additionally, GM composition was more similar between pairs sampled within the minor breeding season than in the major breeding season. The more relaxed territory boundaries in the minor breeding season and possibly fewer seasonal changes due to a shorter minor season, as well as fewer breeding attempts, could explain this, as groups are likely to share more of their geographic range and diet and, hence, a more similar GM (Komdeur 1992, 2001).

In conclusion, our study has been able to separate the effect of sharing habitat from the effect of close social interactions (within cooperative breeding) in shaping the GM of a wild vertebrate. Importantly, we show that different components of the GM are differentially affected by such social interactions: anaerobic microbes are more likely to be transmitted through the cooperative breeding behaviours. Further research is needed to determine whether this elevated sharing of specific microbes due to cooperative breeding is beneficial or detrimental to host fitness.

## Author Contributions

C.Z.L. and D.S.R. conceived the study. C.Z.L. performed the research and analysed the data with input and advice from D.S.R. S.F.W., T.B., J.K., F.H., H.L.D. and D.S.R. supervised the research and acquired funding. C.Z.L. wrote the paper with input from D.S.R. and then all co‐authors. All authors contributed to the review and editing of the paper.

## Funding

C.Z.L. was funded by the UK Biotechnology and Biological Sciences Research Council (BBSRC) Norwich Research Park Biosciences Doctoral Training Partnership (Grant number BB/T008717/1). D.S.R. and H.L.D. were funded by a Natural Environment Research Council (NERC) grant (NE/S010939/1). S.F.W. was funded by a Leverhulme Trust Early Career Fellowship (ECF‐2023‐433). F.H. was supported by the European Research Council H2020 StG (erc‐stg‐948219, EPYC), BBSRC Institute Strategic Programme Food Microbiome and Health (BB/X011054/1, BBS/E/F/000PR13631), Earlham Institute ISP Decoding Biodiversity (BBX011089/1, BBS/E/ER/230002A and BBS/E/ER/230002B). J.K. and D.S.R. were funded by Dutch Science Council grant (ALW NWO Grant No. ALWOP.531), J.K. was funded by NWO TOP grant 854.11.003 and NWO VICI 823.01.014, also from Dutch Science Council. H.L.D. was funded by a Rosalind Franklin Fellowship from the University of Groningen.

## Ethics Statement

Fieldwork was carried out in accordance with local ethical regulations and agreements (UEA ethics approval ID ETH2223‐0665). The Seychelles Department of Environment and the Seychelles Bureau of Standards approved the fieldwork (permit number A0157) and export of samples.

## Consent

The authors have nothing to report.

## Conflicts of Interest

The authors declare no conflicts of interest.

## Supporting information


**Data S1:** mec70304‐sup‐0001‐Supinfo.docx.

## Data Availability

All 16S sequencing data used in this study can be accessed from the European Nucleotide Archive (ENA) database under the study accession numbers PRJEB45408 (samples from 2017 to 2018), PRJEB47095 (from 2019 to 2020) and PRJEB67634 (from 2021 to 2022). *Code availability*: The associated metadata is provided on the GitHub page: https://github.com/Chuen‐Lee/CooperativeBreeding_GM.
