# Supplementary material for: Social Structure and Interactions Differentially Shape Aerotolerant and Anaerobic Gut Microbiomes in a Cooperative Breeding Species
Source: Mol Ecol. 2026 Apr 10;35(7):e70304. doi: 10.1111/mec.70304 (PMC13066922; doi:10.1111/mec.70304)
Supplement: Supplementary file 1 — Data S1: mec70304‐sup‐0001‐Supinfo.docx. [file MEC-35-e70304-s001.docx]

**Supplementary material:**

**Social structure and interactions differentially shape aerotolerant and anaerobic gut microbiomes in a cooperative breeding species**

**Helpers share anaerobic gut microbes**

Chuen Zhang Lee^1,2*^, Sarah F. Worsley^1^, Terry Burke^3^, Jan Komdeur^4^, Falk Hildebrand^5,6^, Hannah L. Dugdale^4^, David S. Richardson^1,7*^

^1^ School of Biological Sciences, University of East Anglia, Norfolk, United Kingdom

^2^ Centre for Microbial Interactions, Norwich Research Park, Norwich, Norfolk, UK

^3^ Ecology and Evolutionary Biology, School of Biosciences, University of Sheffield, Sheffield, United Kingdom

^4^ Groningen Institute for Evolutionary Life Sciences (GELIFES), University of Groningen, Groningen, The Netherlands

^5^ Quadram Institute Biosciences, Norwich Research Park, Norfolk, United Kingdom

^6^ Earlham Institute, Norwich Research Park, Norfolk, United Kingdom

^7^ Nature Seychelles, Roche Caiman, Mahé, Republic of Seychelles, Seychelles

*Correspondence: david.richardson@uea.ac.uk, chuen.lee@uea.ac.uk

**Supplementary metadata**

Table SM1. Summary sample metadata of Seychelles warbler gut microbiome pairwise comparisons for each categorical and continuous variable.

| Categorical variables | Levels | Number of pairwise comparisons | Number of unique samples | | |
| --- | --- | --- | --- | --- | --- |
| Breeding groups |  |  |  |  | |
|  | Between groups | 27542 | 648 | | |
|  | Within groups | 279 | 322 | | |
| Social status categories |  |  |  |  | |
|  | Dom-Dom | 68 | 121 | | |
|  | Dom-Help | 50 | 69 | | |
|  | Dom-Sub | 109 | 156 | | |
|  | Help-Sub | 18 | 21 | | |
|  | Sub-Sub | 34 | 46 | | |
| Sample year |  |  |  | |  |
|  | 2017 | 2750 | 75 | | |
|  | 2018 | 7542 | 147 | | |
|  | 2019 | 3177 | 111 | | |
|  | 2020 | 1886 | 62 | | |
|  | 2021 | 9019 | 135 | | |
|  | 2022 | 3447 | 118 | | |
| Season |  |  |  | |  |
|  | Major | 21215 | 426 | | |
|  | Minor | 6606 | 222 | | |
| Sex |  |  |  | |  |
|  | Same | 14219 | 648 | | |
|  | Different | 13602 | 648 | | |
| Shared nest at hatch |  |  |  | |  |
|  | No | 24946 | 647 | | |
|  | Yes | 2875 | 592 | | |
| Continuous variables | Minimum | Maximum | Average | | SD |
| Age difference | 0 | 16.9 | 3.1 | | 3.2 |
| Time of day | 0 | 98 | 24.6 | | 19.8 |
| Time in season | 0 | 151 | 31.9 | | 29.7 |
| Relatedness | 0 | 0.773 | 0.035 | | 0.067 |

**Supplementary Tables and Figures**

Table S1. A linear mixed effect model investigating the relationship between breeding group membership and gut microbiome ASV richness similarity in pairs of Seychelles warblers (N = 27,821 pairwise comparisons across 648 samples from 345 individual birds). Significant terms (P <0.05) are indicated in bold. Reference categories for categorical variables were the first term in brackets. Time of day was measured as minutes apart, and time in season was measured as days apart.

| **Characteristic** | **Beta** | **SE***^1^* | **Statistic** | **df** | **p-value** |
| --- | --- | --- | --- | --- | --- |
| **(Intercept)** | **-125.7** | **9.14** | **-13.8** | **13.4** | **<0.001** |
| Breeding group (Between/Within) | -0.995 | 6.43 | -0.155 | 27,528 | 0.877 |
| Age difference | -0.067 | 0.308 | -0.217 | 26,377 | 0.828 |
| Sex (same/different) | -0.636 | 1.21 | -0.527 | 27,537 | 0.598 |
| Season (major/minor) | -7.117 | 3.76 | -1.89 | 2,446 | 0.059 |
| Time of day | -0.003 | 0.004 | -0.675 | 27,669 | 0.500 |
| **Time in season** | **-0.157** | **0.035** | **-4.50** | **27,719** | **<0.001** |
| Relatedness | 6.394 | 9.63 | 0.664 | 27,553 | 0.507 |
| Shared nest at hatch (no/yes) | 2.631 | 2.83 | 0.931 | 27,372 | 0.352 |
| **Random** | **27,821 observations** | | | **Variance** | |
| Multi membership ID (Intercept) |  | 345 groups | |  | 49.49 |
| Sample Year (Intercept) |  | 6 years | |  | 17.19 |
| Residual |  |  |  |  | 97.56 |

Table S2. A linear mixed effect model (lmer) investigating the relationship between the social status categories of pairs of Seychelles warblers within breeding groups and the gut microbiome ASV richness similarity between them (N = 279 pairwise comparisons across 322 samples from 204 individual birds). Significant terms (P <0.05) are indicated in bold. Reference categories for categorical variables were the first term in brackets. Time of day was measured as minutes apart, and time in season was measured as days apart.

| **Characteristic** | **Beta** | **SE***^1^* | **Statistic** | **df** | **p-value** |
| --- | --- | --- | --- | --- | --- |
| **(Intercept)** | **-128.9** | **31.2** | **-4.13** | **107** | **<0.001** |
| Individual Status Pair |  |  |  |  |  |
| Dom - Dom | — | — | — |  |  |
| Dom - Help | 13.04 | 31.0 | 0.420 | 217 | 0.675 |
| Dom - Sub | -29.20 | 28.5 | -1.02 | 200 | 0.308 |
| Help - Sub | -4.601 | 41.2 | -0.112 | 163 | 0.911 |
| Sub - Sub | 46.08 | 34.6 | 1.33 | 193 | 0.185 |
| Age difference | -2.380 | 3.20 | -0.743 | 136 | 0.459 |
| Sex (same/different) | -2.730 | 18.6 | -0.147 | 243 | 0.884 |
| Season (major/minor) | -11.05 | 23.1 | -0.479 | 43.8 | 0.635 |
|  |  |  |  |  |  |
| Time of day | 0.099 | 0.077 | 1.29 | 262 | 0.199 |
| Time in season | -0.176 | 0.441 | -0.398 | 267 | 0.691 |
| Relatedness | -41.71 | 44.4 | -0.940 | 187 | 0.348 |
| Shared nest at hatch (no/yes) | 42.05 | 21.7 | 1.94 | 243 | 0.053 |
| **Random** |  | **279 observations** | |  | **Variance** |
| Multi membership ID | (Intercept) | 204 groups | |  | 47.05 |
| Sample Year | (Intercept) | 6 years | |  | 18.09 |
| Residual |  |  |  |  | 109.5 |

Table S3. A linear mixed effect model (lmer) investigating the relationship between the social status categories of pairs of Seychelles warblers within breeding groups and the gut microbiome ASV Shannon diversity similarity between them (N = 279 pairwise comparisons across 322 samples from 204 individual birds). Significant terms (P <0.05) are in bold. Reference categories for categorical variables were the first term in brackets. Time of day was measured as minutes apart, and time in season was measured as days apart.

| **Characteristic** | **Beta** | **SE***^1^* | **Statistic** | **df** | **p-value** |
| --- | --- | --- | --- | --- | --- |
| **(Intercept)** | **-1.425** | **0.230** | **-6.18** | **78.3** | **<0.001** |
| Individual Status Pair |  |  |  |  |  |
| Dom - Dom | — | — | — |  |  |
| **Dom - Help** | **0.542** | **0.226** | **2.40** | **219** | **0.017** |
| Dom - Sub | 0.200 | 0.208 | 0.963 | 204 | 0.337 |
| Help - Sub | 0.288 | 0.303 | 0.950 | 169 | 0.344 |
| Sub - Sub | 0.359 | 0.254 | 1.42 | 194 | 0.159 |
| Age difference | -0.012 | 0.024 | -0.510 | 142 | 0.611 |
| Sex (same/different) | -0.077 | 0.135 | -0.570 | 243 | 0.569 |
| Season (major/minor) | -0.075 | 0.173 | -0.436 | 46.3 | 0.665 |
|  |  |  |  |  |  |
| Time of day | 0.001 | 0.001 | 1.37 | 265 | 0.170 |
| Time in season | <0.001 | 0.003 | 0.059 | 264 | 0.953 |
| Relatedness | -0.613 | 0.324 | -1.89 | 191 | 0.060 |
| Shared nest at hatch (no/yes) | 0.129 | 0.157 | 0.820 | 247 | 0.413 |
| **Random** |  | **279 observations** | |  | **Variance** |
| Multi membership ID | (Intercept) | 204 groups | |  | 0.376 |
| Sample Year | (Intercept) | 6 years | |  | 0.166 |
| Residual |  |  |  |  | 0.762 |

Table S4. Pairwise comparison of social status categories of pairs of Seychelles warblers within breeding groups and the gut microbiome ASV Shannon diversity similarity between them using Tukey method p-values (from Table S2; N = 279 pairwise comparisons across 322 samples from 204 individual birds). Significant terms (P <0.05) are indicated in bold.

| **Contrast** | **Estimate** | **SE** | **df** | **t.ratio** | **p.value** |
| --- | --- | --- | --- | --- | --- |
| Dom-Dom vs Dom-Help | 0.542 | 0.229 | 217 | 2.364 | 0.129 |
| Dom-Dom vs Dom-Sub | 0.200 | 0.210 | 202 | 0.954 | 0.875 |
| Dom-Dom vs Help-Sub | 0.288 | 0.307 | 166 | 0.938 | 0.882 |
| Dom-Dom vs Sub-Sub | 0.359 | 0.260 | 192 | 1.384 | 0.639 |
| Dom-Help vs Dom-Sub | -0.342 | 0.190 | 212 | -1.805 | 0.374 |
| Dom-Help vs Help-Sub | -0.255 | 0.291 | 192 | -0.876 | 0.906 |
| Dom-Help vs Sub-Sub | -0.183 | 0.267 | 214 | -0.687 | 0.959 |
| Dom-Sub vs Help-Sub | 0.087 | 0.282 | 177 | 0.310 | 0.998 |
| Dom-Sub vs Sub-Sub | 0.159 | 0.247 | 206 | 0.642 | 0.968 |
| Help-Sub vs Sub-Sub | 0.072 | 0.333 | 196 | 0.215 | 1.000 |

Table S5. A linear mixed effect model (lmer) investigating the relationship between social status pair categories within a breeding group and GM composition similarity in Seychelles warblers (N = 279 pairwise comparisons across 322 samples from 204 individual birds). Significant terms (P <0.05) are indicated in bold. Reference categories for categorical variables were the first term in brackets. Time of day was measured as minutes apart, and time in season was measured as days apart.

| **Characteristic** | **Beta** | **SE** | **Statistic** | **df** | **p-value** |
| --- | --- | --- | --- | --- | --- |
| **(Intercept)** | **-75.42** | **3.30** | **-22.8** | **24.2** | **<0.001** |
| Individual Status Pair |  |  |  |  |  |
| Dom - Dom | — | — | — |  |  |
| Dom - Help | -0.841 | 2.91 | -0.289 | 237 | 0.773 |
| Dom - Sub | -2.150 | 2.69 | -0.799 | 228 | 0.425 |
| Help - Sub | -2.380 | 4.02 | -0.592 | 198 | 0.555 |
| Sub - Sub | -2.352 | 3.35 | -0.702 | 209 | 0.483 |
| Age difference | -0.451 | 0.322 | -1.40 | 174 | 0.163 |
| Sex (same/different) | -0.199 | 1.69 | -0.117 | 249 | 0.907 |
| Season (major/minor) | 3.977 | 2.39 | 1.66 | 92.6 | 0.100 |
| Time of day | -0.001 | 0.007 | -0.222 | 254 | 0.824 |
| Time in season | 0.002 | 0.037 | 0.065 | 222 | 0.948 |
| Relatedness | -3.252 | 4.23 | -0.768 | 220 | 0.443 |
| Shared nest at hatch (no/yes) | 1.368 | 1.95 | 0.701 | 262 | 0.484 |
| **Random** |  | **279 observations** | |  | **Variance** |
| Multi membership ID | (Intercept) | 204 groups | |  | 6.449 |
| Sample Year | (Intercept) | 6 years | |  | 4.151 |
| Residual |  |  |  |  | 8.037 |

Table S6. A linear mixed effect model (lmer) investigating the relationship between individual status pairs and **aerotolerant** GM composition similarity of Seychelles warblers (N = 279 pairwise comparisons across 322 samples from 204 individual birds). Significant terms (P <0.05) are indicated in bold. Reference categories for categorical variables were the first term in brackets. Time of day was measured as minutes apart, and time in season was measured as days apart.

| **Characteristic** | **Beta** | **SE***^1^* | **Statistic** | df | **p-value** |
| --- | --- | --- | --- | --- | --- |
| **(Intercept)** | **-41.42** | **1.86** | **-22.2** | **18.7** | **<0.001** |
| Individual Status Pair |  |  |  |  |  |
| Dom - Dom | — | — | — |  |  |
| Dom - Help | -0.831 | 1.68 | -0.495 | 232 | 0.621 |
| Dom - Sub | -1.804 | 1.56 | -1.16 | 227 | 0.248 |
| Help - Sub | -3.017 | 2.33 | -1.29 | 198 | 0.197 |
| Sub - Sub | -1.902 | 1.88 | -1.01 | 207 | 0.313 |
| **Age difference** | **-0.245** | **0.074** | **-3.29** | **212** | **<0.001** |
| Sex (same/different) | -1.102 | 0.973 | -1.13 | 245 | 0.258 |
| Season (major/minor) | 1.833 | 1.40 | 1.31 | 116 | 0.192 |
| Time of day | <0.001 | 0.004 | -0.040 | 254 | 0.968 |
| Time in season | -0.001 | 0.021 | -0.055 | 224 | 0.956 |
| Relatedness | -2.428 | 2.45 | -0.991 | 221 | 0.323 |
| Shared nest at hatch (no/yes) | -0.389 | 1.12 | -0.346 | 261 | 0.729 |
| **Random** |  | **279 observations** | |  | **Variance** |
| Multi membership ID | (Intercept) | 204 groups | |  | 3.785 |
| Sample Year | (Intercept) | 6 years | |  | 2.638 |
| Residual |  |  |  |  | 4.588 |

Table S7. A pairwise comparison with Tukey method p-values of the relationship between individual status pairs and **anaerobic** GM composition dissimilarity of Seychelles warblers (from Table S10) (N = 279 pairwise comparisons across 322 samples from 204 individual birds). Significant terms (P <0.05) are indicated in bold

| **Contrast** | **Estimate** | **SE** | **df** | **t.ratio** | **p.value** |
| --- | --- | --- | --- | --- | --- |
| Dom-Dom vs Dom-Help | -0.661 | 1.240 | 202 | -0.534 | 0.984 |
| Dom-Dom vs Dom-Sub | -2.231 | 1.150 | 186 | -1.945 | 0.297 |
| Dom-Dom vs Help-Sub | -3.483 | 1.650 | 150 | -2.110 | 0.221 |
| Dom-Dom vs Sub-Sub | -3.319 | 1.360 | 179 | -2.434 | 0.111 |
| Dom-Help vs Dom-Sub | -1.570 | 1.030 | 205 | -1.521 | 0.550 |
| Dom-Help vs Help-Sub | -2.822 | 1.580 | 182 | -1.790 | 0.383 |
| Dom-Help vs Sub-Sub | -2.658 | 1.440 | 204 | -1.841 | 0.353 |
| Dom-Sub vs Help-Sub | -1.252 | 1.530 | 169 | -0.819 | 0.925 |
| Dom-Sub vs Sub-Sub | -1.088 | 1.310 | 200 | -0.829 | 0.921 |
| Help-Sub vs Sub-Sub | 0.164 | 1.790 | 190 | 0.092 | 1.000 |

Table S8. A linear mixed effect model (lmer) investigating the relationship between the **anaerobic** GM composition similarity of nest-sharing pairs of Seychelles warblers compared to non-nest-sharing pairs (N = 279 pairwise comparisons across 320 samples from 204 individual birds). Significant terms (P <0.05) are indicated in bold. Reference categories for categorical variables were the first term in brackets. Time of day was measured as minutes apart, and time in season was measured as days apart.

| **Characteristic** | **Beta** | **SE***^1^* | **Statistic** | **df** | **p-value** |
| --- | --- | --- | --- | --- | --- |
| **(Intercept)** | **-22.970** | **1.141** | **-20.135** | **30** | **<0.001** |
| **Nest sharing group (yes/no)** | **-2.317** | **0.778** | **-2.977** | **197** | **0.003** |
| Age difference | 0.015 | 0.066 | 0.223 | 261 | 0.824 |
| Sex (same/different) | 0.490 | 0.694 | 0.706 | 240 | 0.481 |
| Season (major/minor) | 0.157 | 1.040 | 0.151 | 84 | 0.880 |
| Time of day | -0.001 | 0.003 | -0.463 | 254 | 0.644 |
| Time in season | <0.001 | 0.018 | -0.024 | 263 | 0.981 |
| Relatedness | 1.622 | 1.754 | 0.925 | 206 | 0.356 |
| Shared nest at hatch (no/yes) | -0.096 | 0.730 | -0.131 | 242 | 0.896 |
| **Random** | | **274 observations** | | **Variance** | |
| Multi membership ID | (Intercept) |  | 204 groups | 3.432 | |
| Sample Year | (Intercept) |  | 6 years | 2.180 | |
| Residual |  |  |  |  |  |

Table S9. The aerotolerance status of the bacterial genera from the gut microbiome of the Seychelles warbler, assigned using Google Gemini 2.0 on 21^st^ January 2025. A total of 891 out of 1111 genera could be assigned. The correspondence with Google Gemini were validated in two ways: manual verification of a random subset of 80 genera using Bergey’s Manual (Gemini vs. Manual assignment; 96.3% correspondence), and cross-checking of 145 genera against the assignments reported by Raulo et al. (2024), which showed 94.5% correspondence.

| Genus | Gemini | Gemini vs. Manual | Gemini vs. Raulo et al. (2024) |
| --- | --- | --- | --- |
| [Clostridium] innocuum group | Anaerobic | - | - |
| [Eubacterium] brachy group | Anaerobic | Correct | Correct |
| [Eubacterium] coprostanoligenes group | Anaerobic | - | - |
| [Eubacterium] fissicatena group | Anaerobic | - | - |
| [Eubacterium] nodatum group | Anaerobic | - | Correct |
| [Eubacterium] xylanophilum group | Anaerobic | - | Correct |
| [Ruminococcus] torques group | Anaerobic | - | Correct |
| 1174-901-12 | Unknown | - | - |
| 1921-2 | Unknown | - | - |
| 1959-1 | Unknown | - | - |
| 3PJM14 | Unknown | - | - |
| A2 | Anaerobic | - | - |
| Acetobacter | Aerotolerant | - | - |
| Acholeplasma | Aerotolerant | - | - |
| Achromobacter | Aerotolerant | - | Correct |
| Acidaminococcus | Anaerobic | - | - |
| Acidibacter | Aerotolerant | - | Correct |
| Acidicaldus | Aerotolerant | - | - |
| Acidiphilium | Aerotolerant | - | Correct |
| Acidipropionibacterium | Anaerobic | - | - |
| Acidothermus | Aerotolerant | - | - |
| Acinetobacter | Aerotolerant | - | Correct |
| Actibacterium | Aerotolerant | - | - |
| Actinoallomurus | Aerotolerant | - | - |
| Actinoalloteichus | Aerotolerant | - | - |
| Actinobacillus | Aerotolerant | - | - |
| actinobacterium BGR 88 | Unknown | - | - |
| Actinocatenispora | Aerotolerant | - | - |
| Actinocorallia | Aerotolerant | - | - |
| Actinokineospora | Aerotolerant | - | - |
| Actinomadura | Aerotolerant | - | - |
| Actinomyces | Anaerobic | - | - |
| Actinomycetospora | Aerotolerant | - | - |
| Actinophytocola | Aerotolerant | - | - |
| Actinoplanes | Aerotolerant | Correct | Correct |
| Actinopolymorpha | Aerotolerant | - | - |
| Actinopolyspora | Aerotolerant | - | - |
| Actinospica | Aerotolerant | - | - |
| Actinotalea | Aerotolerant | - | - |
| Acuticoccus | Aerotolerant | - | - |
| Adhaeribacter | Aerotolerant | - | Correct |
| ADurb.Bin063-1 | Unknown | - | - |
| Aequorivita | Aerotolerant | - | - |
| Aeromicrobium | Aerotolerant | - | - |
| Aeromonas | Aerotolerant | - | Correct |
| Aerosphaera | Aerotolerant | - | - |
| Aestuariimicrobium | Aerotolerant | - | - |
| Afifella | Aerotolerant | - | - |
| Afipia | Aerotolerant | - | - |
| Agaricicola | Aerotolerant | - | - |
| Agrococcus | Aerotolerant | Correct | - |
| Agromyces | Aerotolerant | - | Correct |
| Akkermansia | Anaerobic | Correct | - |
| AKYG587 | Unknown | - | - |
| Alcaligenes | Aerotolerant | - | - |
| Algisphaera | Aerotolerant | - | - |
| Algoriphagus | Aerotolerant | - | - |
| Alicycliphilus | Aerotolerant | - | - |
| Aliifodinibius | Anaerobic | - | - |
| Aliihoeflea | Aerotolerant | - | - |
| Alishewanella | Aerotolerant | - | - |
| Alistipes | Anaerobic | Correct | Correct |
| Alkaliphilus | Aerotolerant | - | - |
| Alkanindiges | Aerotolerant | - | - |
| Allochromatium | Anaerobic | - | - |
| Alloprevotella | Anaerobic | - | Correct |
| Allorhizobium-Neorhizobium-Pararhizobium-Rhizobium | Aerotolerant | - | Correct |
| alphaI cluster | Unknown | - | - |
| Altererythrobacter | Aerotolerant | - | Correct |
| Alterococcus | Aerotolerant | - | - |
| Alteromonas | Aerotolerant | - | - |
| Amaricoccus | Aerotolerant | - | Correct |
| Ambiguous_taxa | Unknown | - | - |
| Aminobacter | Aerotolerant | - | Correct |
| Ammoniphilus | Aerotolerant | - | - |
| Amnibacterium | Aerotolerant | Correct | - |
| Amphibacillus | Aerotolerant | Correct | - |
| Amphiplicatus | Aerotolerant | - | - |
| Amycolatopsis | Aerotolerant | - | - |
| Anaerobacillus | Anaerobic | - | - |
| Anaerobiospirillum | Anaerobic | - | - |
| Anaerobium | Anaerobic | - | - |
| Anaerococcus | Anaerobic | - | - |
| Anaerocolumna | Anaerobic | - | - |
| Anaerofilum | Anaerobic | - | - |
| Anaerofustis | Anaerobic | Correct | - |
| Anaeromyxobacter | Aerotolerant | - | - |
| Anaeroplasma | Anaerobic | - | Correct |
| Anaerosinus | Anaerobic | - | - |
| Anaerosolibacter | Anaerobic | - | - |
| Anaerosporobacter | Anaerobic | - | Correct |
| Anaerostipes | Anaerobic | - | Correct |
| Anaerotruncus | Anaerobic | - | - |
| Anaerovibrio | Anaerobic | - | - |
| Anaerovorax | Anaerobic | Correct | - |
| Ancylobacter | Aerotolerant | - | - |
| Angustibacter | Anaerobic | - | - |
| Anoxybacillus | Aerotolerant | - | - |
| Anthococcus | Aerotolerant | - | - |
| Antricoccus | Aerotolerant | - | - |
| Apibacter | Aerotolerant | - | - |
| Aquabacterium | Aerotolerant | - | - |
| Aquamicrobium | Aerotolerant | - | - |
| Aquicella | Aerotolerant | - | - |
| Aquipuribacter | Aerotolerant | - | - |
| Aquisphaera | Aerotolerant | - | - |
| Aquitalea | Aerotolerant | - | - |
| Arcobacter | Aerotolerant | - | - |
| Arcticibacter | Aerotolerant | - | - |
| Arenimonas | Aerotolerant | - | Correct |
| Arhodomonas | Aerotolerant | - | - |
| Aridibacter | Aerotolerant | - | - |
| Armatimonas | Aerotolerant | - | - |
| Arsenicicoccus | Aerotolerant | - | - |
| Arsenophonus | Aerotolerant | - | - |
| Arthrobacter | Aerotolerant | - | Correct |
| Asaia | Aerotolerant | - | - |
| Asanoa | Aerotolerant | Correct | - |
| Asticcacaulis | Aerotolerant | - | - |
| AT-s3-44 | Unknown | - | - |
| Atopobium | Anaerobic | - | - |
| Atopostipes | Aerotolerant | - | - |
| Aureimonas | Aerotolerant | - | - |
| Azoarcus | Anaerobic | - | - |
| Azospira | Anaerobic | - | - |
| Azospirillum | Aerotolerant | - | - |
| Bacillus | Aerotolerant | Correct | - |
| bacterium Ellin6529 | Unknown | - | - |
| bacterium Ellin6537 | Unknown | - | - |
| bacterium Ellin6543 | Unknown | - | - |
| bacterium enrichment culture clone Anammox_49 | Anaerobic | - | - |
| bacterium enrichment culture clone auto112_4W | Unknown | - | - |
| bacterium LWQ8 | Unassigned | - | - |
| bacterium WWH38 | Unassigned | - | - |
| bacterium WX65 | Unknown | - | - |
| bacterium YC-ZSS-LKJ159 | Unknown | - | - |
| bacterium YC-ZSS-LKJ66 | Unknown | - | - |
| Bacteroides | Anaerobic | Correct | Correct |
| Baia | Aerotolerant | - | - |
| Bartonella | Aerotolerant | - | - |
| Bauldia | Aerotolerant | - | Correct |
| BD1-7 clade | Unknown | - | - |
| Bdellovibrio | Aerotolerant | - | - |
| Belnapia | Aerotolerant | - | - |
| Bhargavaea | Aerotolerant | - | - |
| Bifidobacterium | Anaerobic | - | Correct |
| Bilophila | Anaerobic | - | Correct |
| BIyi10 | Unknown | - | - |
| Blastocatella | Aerotolerant | - | Correct |
| Blastochloris | Anaerobic | - | - |
| Blastococcus | Aerotolerant | - | - |
| Blastomonas | Aerotolerant | - | - |
| Blastopirellula | Aerotolerant | - | Correct |
| Blattabacterium | Unknown | - | - |
| Blattella germanica (German cockroach) | Unknown | - | - |
| Blautia | Anaerobic | - | Correct |
| Bogoriella | Aerotolerant | - | - |
| Bordetella | Aerotolerant | - | - |
| Bosea | Aerotolerant | - | - |
| Brachybacterium | Aerotolerant | Correct | - |
| Brachyspira | Anaerobic | - | - |
| Bradyrhizobium | Aerotolerant | - | Correct |
| Brevibacillus | Aerotolerant | - | - |
| Brevibacterium | Aerotolerant | Correct | - |
| Brevundimonas | Aerotolerant | - | Correct |
| Breznakia | Anaerobic | - | - |
| Brooklawnia | Aerotolerant | - | - |
| Bryobacter | Aerotolerant | - | Correct |
| Burkholderia-Caballeronia-Paraburkholderia | Aerotolerant | - | - |
| Butyricicoccus | Anaerobic | - | Correct |
| Butyricimonas | Anaerobic | - | - |
| Bythopirellula | Aerotolerant | - | - |
| C1-B045 | Unknown | - | - |
| C39 | Unknown | - | - |
| Caenimonas | Aerotolerant | - | Correct |
| Camelimonas | Aerotolerant | - | - |
| Campylobacter | Aerotolerant | - | - |
| candidate division SR1 bacterium MGEHA | Unassigned | - | - |
| candidate division TM7 bacterium JGI 0001002-L20 | Unassigned | - | - |
| candidate division TM7 bacterium LY2 | Unassigned | - | - |
| Candidatus Actinomarina | Unknown | - | - |
| Candidatus Alysiosphaera | Unknown | - | - |
| Candidatus Anammoximicrobium | Anaerobic | - | - |
| Candidatus Arthromitus | Unknown | - | - |
| Candidatus Bacilloplasma | Unknown | - | - |
| Candidatus Bealeia | Unknown | - | - |
| Candidatus Berkiella | Unknown | - | - |
| Candidatus Blochmannia | Unknown | - | - |
| Candidatus Cardinium | Unknown | - | - |
| Candidatus Chloroploca | Anaerobic | - | - |
| Candidatus Competibacter | Unknown | - | - |
| Candidatus Dichloromethanomonas | Anaerobic | - | - |
| Candidatus Entotheonella | Unknown | - | - |
| Candidatus Finniella | Unknown | - | - |
| Candidatus Jidaibacter | Unknown | - | - |
| Candidatus Kinetoplastibacterium | Unknown | - | - |
| Candidatus Koribacter | Aerotolerant | - | - |
| Candidatus Megaira | Unknown | - | - |
| Candidatus Moranella | Unknown | - | - |
| Candidatus Nostocoida | Anaerobic | - | - |
| Candidatus Omnitrophus | Unknown | - | - |
| Candidatus Ovatusbacter | Unknown | - | - |
| Candidatus Paracaedibacter | Unknown | - | - |
| Candidatus Portiera | Unknown | - | - |
| Candidatus Rokubacteria bacterium CSP1-6 | Unknown | - | - |
| Candidatus Rosenkranzia | Unknown | - | - |
| Candidatus Saccharibacteria bacterium RAAC3_TM7_1 | Unknown | - | - |
| Candidatus Saccharibacteria bacterium UB2523 | Unknown | - | - |
| Candidatus Saccharimonas | Unknown | - | - |
| Candidatus Soleaferrea | Anaerobic | - | - |
| Candidatus Solibacter | Aerotolerant | - | - |
| Candidatus Sulcia | Unknown | - | - |
| Candidatus Tenderia | Unknown | - | - |
| Candidatus Tremblaya | Unknown | - | - |
| Candidatus Udaeobacter | Unknown | - | - |
| Candidatus Uzinura | Unknown | - | - |
| Candidatus Vestibaculum | Unknown | - | - |
| Candidatus Xiphinematobacter | Unknown | - | - |
| Caproiciproducens | Anaerobic | - | Correct |
| Carnimonas | Aerotolerant | - | - |
| Carnobacterium | Aerotolerant | - | Correct |
| Castellaniella | Aerotolerant | - | - |
| Catabacter | Anaerobic | - | - |
| Catellatospora | Aerotolerant | - | - |
| Catellicoccus | Aerotolerant | - | - |
| Caulobacter | Aerotolerant | - | - |
| Celeribacter | Aerotolerant | - | - |
| Cellulomonas | Aerotolerant | Correct | - |
| Cellulosilyticum | Anaerobic | Correct | Correct |
| Cellulosimicrobium | Aerotolerant | - | - |
| Cellvibrio | Aerotolerant | - | - |
| Cetobacterium | Anaerobic | - | - |
| Chalicogloea CCALA 975 | Aerotolerant | - | - |
| Chelativorans | Aerotolerant | - | - |
| Chelatococcus | Aerotolerant | - | - |
| Chelonobacter | Aerotolerant | - | - |
| Chitinimonas | Aerotolerant | - | - |
| Chitinophaga | Aerotolerant | - | - |
| CHKCI002 | Unknown | - | - |
| Chloroflexi bacterium JEA33 | Anaerobic | - | - |
| Chlorogloeopsis PCC-7518 | Aerotolerant | - | - |
| Chloronema | Anaerobic | - | - |
| Chondromyces | Aerotolerant | - | - |
| Christensenella | Anaerobic | - | - |
| Christensenellaceae R-7 group | Anaerobic | Correct | - |
| Chroococcidiopsis PCC 7203 | Aerotolerant | - | - |
| Chroococcidiopsis PCC-6712 | Aerotolerant | - | - |
| Chroococcidiopsis SAG 2023 | Aerotolerant | - | - |
| Chryseobacterium | Aerotolerant | - | Correct |
| Chryseolinea | Aerotolerant | - | Correct |
| Chthoniobacter | Aerotolerant | Correct | - |
| Chthonobacter | Aerotolerant | - | - |
| Chthonomonas | Aerotolerant | - | - |
| Chujaibacter | Aerotolerant | - | - |
| CL500-29 marine group | Unknown | - | - |
| CL500-3 | Unknown | - | - |
| Cloacibacillus | Aerotolerant | - | - |
| Cloacibacterium | Anaerobic | - | - |
| Clostridiisalibacter | Anaerobic | - | - |
| Clostridioides | Anaerobic | - | - |
| Clostridium sensu stricto 1 | Anaerobic | Correct | - |
| Clostridium sensu stricto 10 | Anaerobic | - | - |
| Clostridium sensu stricto 11 | Anaerobic | - | - |
| Clostridium sensu stricto 12 | Anaerobic | - | - |
| Clostridium sensu stricto 13 | Anaerobic | - | - |
| Clostridium sensu stricto 18 | Anaerobic | - | - |
| Clostridium sensu stricto 2 | Anaerobic | - | - |
| Clostridium sensu stricto 3 | Anaerobic | - | - |
| Clostridium sensu stricto 5 | Anaerobic | - | - |
| Clostridium sensu stricto 6 | Anaerobic | - | - |
| Clostridium sensu stricto 8 | Anaerobic | - | - |
| Cnuella | Aerotolerant | - | - |
| Cohnella | Aerotolerant | - | Correct |
| Collinsella | Anaerobic | - | - |
| Comamonas | Aerotolerant | - | - |
| Commensalibacter | Aerotolerant | - | - |
| Conexibacter | Aerotolerant | - | - |
| Constrictibacter | Aerotolerant | - | - |
| Coprobacillus | Aerotolerant | - | - |
| Coprococcus 3 | Anaerobic | - | - |
| Coriobacteriaceae UCG-002 | Anaerobic | - | - |
| Corticicoccus | Aerotolerant | - | - |
| Corynebacterium | Aerotolerant | - | Correct |
| Corynebacterium 1 | Aerotolerant | Correct | - |
| Coxiella | Aerotolerant | - | - |
| CPla-4 termite group | Unknown | - | - |
| Craurococcus | Aerotolerant | - | - |
| Croceicoccus | Aerotolerant | - | - |
| Crocinitomix | Aerotolerant | - | - |
| Crossiella | Aerotolerant | - | - |
| Cryomorpha | Aerotolerant | - | - |
| Cryptosporangium | Aerotolerant | Correct | - |
| Cupriavidus | Aerotolerant | - | - |
| Curtobacterium | Aerotolerant | Correct | Correct |
| Curvibacter | Aerotolerant | - | - |
| Cutibacterium | Aerotolerant | - | Correct |
| Cyanobium PCC-6307 | Aerotolerant | - | - |
| Cystobacter | Aerotolerant | - | - |
| Cytophaga | Aerotolerant | - | Correct |
| Dactylosporangium | Aerotolerant | - | - |
| Dechloromonas | Anaerobic | - | - |
| Defluviicoccus | Aerotolerant | - | - |
| Defluviimonas | Aerotolerant | - | - |
| Defluviitaleaceae UCG-011 | Anaerobic | - | - |
| Deinococcus | Aerotolerant | - | - |
| Delftia | Aerotolerant | - | - |
| Demequina | Aerotolerant | Correct | - |
| Dendrosporobacter | Anaerobic | - | - |
| Dermacoccus | Aerotolerant | - | - |
| Dermatophilus | Aerotolerant | - | - |
| Desulfatiferula | Anaerobic | - | - |
| Desulfotomaculum | Anaerobic | - | - |
| Desulfovibrio | Anaerobic | - | Correct |
| Devosia | Aerotolerant | - | - |
| Dietzia | Aerotolerant | Correct | - |
| Dinghuibacter | Aerotolerant | - | Correct |
| Diplorickettsia | Aerotolerant | - | - |
| Dokdonella | Aerotolerant | - | - |
| Domibacillus | Aerotolerant | - | - |
| Dongia | Aerotolerant | - | Correct |
| Dorea | Anaerobic | - | - |
| DTU089 | Anaerobic | - | - |
| Dyadobacter | Aerotolerant | - | - |
| Dyella | Aerotolerant | - | - |
| Dysgonomonas | Anaerobic | Wrong | - |
| EcFYyy-200 | Unknown | - | - |
| Eggerthella | Anaerobic | - | - |
| Eikenella | Aerotolerant | - | - |
| Eisenbergiella | Anaerobic | - | Correct |
| Ellin6055 | Unknown | - | - |
| Ellin6067 | Unknown | - | - |
| Elusimicrobium | Aerotolerant | - | Wrong |
| Empedobacter | Aerotolerant | - | - |
| Emticicia | Aerotolerant | - | - |
| Endobacter | Aerotolerant | - | - |
| endosymbionts6 | Unknown | - | - |
| endosymbionts8 | Unassigned | - | - |
| Enhydrobacter | Aerotolerant | - | - |
| Ensifer | Aerotolerant | - | - |
| Enteractinococcus | Aerotolerant | - | - |
| Enterococcus | Aerotolerant | Correct | Correct |
| Enterorhabdus | Anaerobic | - | Wrong |
| Enterovibrio | Aerotolerant | - | - |
| Entomoplasma | Aerotolerant | - | - |
| Eoetvoesia | Aerotolerant | - | - |
| Epulopiscium | Anaerobic | - | - |
| Erysipelatoclostridium | Anaerobic | Correct | Correct |
| Erysipelothrix | Aerotolerant | - | - |
| Erythrobacter | Aerotolerant | - | - |
| Erythrobacter sp. HME6855 | Aerotolerant | - | - |
| Escherichia-Shigella | Aerotolerant | - | Correct |
| Eubacterium | Anaerobic | Correct | - |
| Euryhalocaulis | Aerotolerant | - | - |
| Euzebya | Aerotolerant | - | - |
| Facklamia | Aerotolerant | - | - |
| Faecalitalea | Anaerobic | - | - |
| Family XIII AD3011 group | Anaerobic | - | - |
| Family XIII UCG-001 | Anaerobic | - | - |
| FCPS473 | Unknown | - | - |
| Fermentimonas | Anaerobic | - | - |
| Ferrovibrio | Aerotolerant | - | - |
| Ferruginibacter | Aerotolerant | - | Correct |
| FFCH5858 | Unknown | - | - |
| FFCH7168 | Unknown | - | - |
| Fibrella | Aerotolerant | - | - |
| Filimonas | Anaerobic | - | - |
| Fimbriiglobus | Aerotolerant | Correct | Correct |
| Flavihumibacter | Aerotolerant | - | - |
| Flavimarina | Aerotolerant | - | - |
| Flavisolibacter | Aerotolerant | - | - |
| Flavitalea | Aerotolerant | - | - |
| Flavobacterium | Aerotolerant | - | Correct |
| Flectobacillus | Aerotolerant | - | - |
| Flexibacter | Aerotolerant | - | - |
| Flexivirga | Aerotolerant | - | - |
| Flindersiella | Aerotolerant | - | - |
| Fluviicola | Aerotolerant | - | - |
| Fodinicola | Aerotolerant | - | - |
| Foliisarcina CENA333 | Aerotolerant | - | - |
| Fonticella | Anaerobic | - | - |
| Fontimonas | Aerotolerant | - | - |
| Formosa | Aerotolerant | - | - |
| Fournierella | Anaerobic | - | - |
| Friedmanniella | Aerotolerant | - | - |
| Frigoribacterium | Aerotolerant | Correct | - |
| Fructobacillus | Aerotolerant | Correct | - |
| Fulvimarina | Aerotolerant | - | - |
| Fusobacterium | Anaerobic | - | - |
| Gaiella | Aerotolerant | - | Correct |
| Gaiella sp. EBR4-RS1 | Aerotolerant | - | - |
| Galbibacter | Aerotolerant | - | - |
| Galbitalea | Aerotolerant | - | - |
| Gallicola | Aerotolerant | - | - |
| Gardnerella | Anaerobic | - | - |
| GCA-900066225 | Anaerobic | - | - |
| GCA-900066575 | Anaerobic | - | - |
| Gelidibacter | Aerotolerant | - | - |
| Gemella | Aerotolerant | - | - |
| Geminicoccus | Aerotolerant | - | - |
| Gemmata | Aerotolerant | Correct | Correct |
| Gemmatimonas | Aerotolerant | - | Correct |
| Gemmatirosa | Aerotolerant | - | - |
| Gemmobacter | Aerotolerant | - | - |
| Geobacillus | Aerotolerant | - | - |
| Geodermatophilus | Aerotolerant | Correct | - |
| Geomicrobium | Aerotolerant | - | - |
| Georgenia | Aerotolerant | - | - |
| Gilliamella | Aerotolerant | - | - |
| Gimesia | Aerotolerant | - | - |
| GKS98 freshwater group | Unknown | - | - |
| Glaciecola | Aerotolerant | - | - |
| Gloeocapsa PCC-7428 | Aerotolerant | - | - |
| Gluconobacter | Aerotolerant | - | - |
| Glutamicibacter | Aerotolerant | - | - |
| Glycomyces | Aerotolerant | - | - |
| Gordonia | Aerotolerant | Correct | - |
| Gordonibacter | Aerotolerant | - | Wrong |
| Gottschalkia | Anaerobic | - | - |
| Gracilibacillus | Aerotolerant | - | - |
| Gramella | Aerotolerant | - | - |
| Granulicella | Aerotolerant | - | - |
| Gryllotalpicola | Aerotolerant | - | - |
| Gulosibacter | Aerotolerant | - | - |
| Haematomicrobium | Aerotolerant | - | - |
| Haemophilus | Aerotolerant | - | - |
| Halalkalibacillus | Aerotolerant | - | - |
| Haliangium | Aerotolerant | - | Correct |
| Haloactinobacterium | Aerotolerant | - | - |
| Haloactinopolyspora | Aerotolerant | - | - |
| Haloactinospora | Aerotolerant | - | - |
| Halobacillus | Aerotolerant | - | - |
| Halobacteriovorax | Aerotolerant | - | - |
| Halodesulfovibrio | Anaerobic | - | - |
| Haloferula | Aerotolerant | - | - |
| Halomonas | Aerotolerant | - | - |
| Haloplasma | Anaerobic | - | - |
| Halovibrio | Aerotolerant | - | - |
| Hamadaea | Aerotolerant | - | - |
| Hansschlegelia | Aerotolerant | - | - |
| Haoranjiania | Aerotolerant | - | - |
| Hathewaya | Anaerobic | - | - |
| Helicobacter | Aerotolerant | - | Correct |
| Hellea | Aerotolerant | - | - |
| Hephaestia | Aerotolerant | - | - |
| Herbaspirillum | Aerotolerant | - | - |
| Herbinix | Anaerobic | - | - |
| Herpetosiphon | Anaerobic | - | - |
| Hirschia | Aerotolerant | - | - |
| Hoeflea | Aerotolerant | - | - |
| Holdemania | Anaerobic | Correct | - |
| Hoyosella | Aerotolerant | - | - |
| HSB OF53-F07 | Unknown | - | - |
| Huakuichenia | Aerotolerant | - | - |
| Humibacter | Aerotolerant | - | - |
| Hungatella | Anaerobic | - | - |
| Hyaloperonospora arabidopsidis | Unknown | - | - |
| Hydrogenispora | Anaerobic | - | - |
| Hydrogenoanaerobacterium | Anaerobic | - | - |
| Hydrogenophaga | Aerotolerant | - | - |
| Hymenobacter | Aerotolerant | - | - |
| Hyphomicrobium | Aerotolerant | - | Correct |
| Iamia | Aerotolerant | - | Correct |
| Idiomarina | Aerotolerant | - | - |
| Ignatzschineria | Aerotolerant | - | - |
| IheB3-7 | Unknown | - | - |
| Ilumatobacter | Aerotolerant | - | Correct |
| IMCC26207 | Unknown | - | - |
| Incertae Sedis | Unknown | - | - |
| Inhella | Aerotolerant | - | - |
| Intestinibacter | Anaerobic | - | - |
| Intestinimonas | Anaerobic | - | Correct |
| IS-44 | Unknown | - | - |
| Isoptericola | Aerotolerant | - | - |
| Isosphaera | Aerotolerant | - | - |
| Jannaschia | Aerotolerant | - | - |
| Jatrophihabitans | Aerotolerant | Correct | - |
| JCM 18997 | Unknown | - | - |
| JdFR-76 | Unknown | - | - |
| Jeotgalibaca | Aerotolerant | - | - |
| Jeotgalicoccus | Aerotolerant | - | - |
| JGI 0001001-H03 | Unknown | - | - |
| Jiangella | Aerotolerant | - | - |
| Jonesia | Aerotolerant | - | - |
| JTB255 marine benthic group | Unknown | - | - |
| Kaistia | Aerotolerant | - | - |
| Ketogulonicigenium | Aerotolerant | - | - |
| Kibdelosporangium | Aerotolerant | - | - |
| Kineococcus | Aerotolerant | Correct | - |
| Kineosporia | Aerotolerant | Correct | - |
| Kocuria | Aerotolerant | - | - |
| Kosakonia | Aerotolerant | - | - |
| Koukoulia | Aerotolerant | - | - |
| Kouleothrix | Anaerobic | - | - |
| Kribbella | Aerotolerant | - | Correct |
| Ktedonobacter | Aerotolerant | - | - |
| Kurthia | Aerotolerant | - | - |
| Kushneria | Aerotolerant | - | - |
| Labrenzia | Aerotolerant | - | - |
| Labrys | Aerotolerant | - | - |
| Lachnoclostridium | Anaerobic | Correct | Correct |
| Lachnoclostridium 10 | Anaerobic | Correct | - |
| Lachnoclostridium 12 | Anaerobic | - | - |
| Lachnoclostridium 5 | Anaerobic | - | - |
| Lachnospira | Anaerobic | - | - |
| Lachnospiraceae NK4A136 group | Anaerobic | - | - |
| Lachnospiraceae UCG-006 | Anaerobic | - | - |
| Lachnospiraceae UCG-007 | Anaerobic | Correct | - |
| Lachnospiraceae UCG-008 | Anaerobic | - | - |
| Lachnospiraceae UCG-009 | Anaerobic | - | - |
| Lachnospiraceae UCG-010 | Anaerobic | Correct | - |
| Lachnotalea | Anaerobic | - | - |
| Lacticigenium | Aerotolerant | - | - |
| Lactobacillus | Aerotolerant | Correct | Correct |
| Lactococcus | Aerotolerant | Correct | Correct |
| Lactonifactor | Anaerobic | - | - |
| Lactovum | Aerotolerant | - | - |
| Lacunisphaera | Aerotolerant | - | - |
| Lamprocystis | Anaerobic | - | - |
| Lampropedia | Aerotolerant | - | - |
| Larkinella | Aerotolerant | - | - |
| Lautropia | Aerotolerant | - | - |
| Lawsonella | Aerotolerant | - | - |
| LD29 | Unknown | - | - |
| Leadbetterella | Aerotolerant | - | - |
| Lechevalieria | Aerotolerant | - | - |
| Legionella | Aerotolerant | - | - |
| Leifsonia | Aerotolerant | Correct | - |
| Lentimicrobium | Aerotolerant | - | - |
| Leptolyngbya ANT.L52.2 | Aerotolerant | - | - |
| Leptolyngbya Es-Yyy1000 | Aerotolerant | - | - |
| Leptolyngbya PCC-6306 | Aerotolerant | - | - |
| Leucobacter | Aerotolerant | Correct | - |
| Leuconostoc | Aerotolerant | Correct | - |
| Lewinella | Aerotolerant | - | - |
| Limibacillus | Aerotolerant | - | - |
| Limnobacter | Aerotolerant | - | - |
| Listeria | Aerotolerant | - | - |
| Litorilinea | Aerotolerant | - | - |
| Longimicrobium | Aerotolerant | - | - |
| Longimonas | Anaerobic | - | - |
| Longispora | Aerotolerant | - | - |
| Loriellopsis LF-B5 | Aerotolerant | - | - |
| Luedemannella | Aerotolerant | - | - |
| Luteibacter | Aerotolerant | - | - |
| Luteimonas | Aerotolerant | - | - |
| Luteitalea | Aerotolerant | - | Correct |
| Luteococcus | Aerotolerant | - | - |
| Luteolibacter | Aerotolerant | - | - |
| Lutispora | Anaerobic | - | - |
| Lysinibacillus | Aerotolerant | - | - |
| Lysinimicrobium | Aerotolerant | - | - |
| Lysobacter | Aerotolerant | - | - |
| Mangrovibacter | Aerotolerant | - | - |
| Marinagarivorans | Aerotolerant | - | - |
| marine metagenome | Unassigned | - | - |
| Marinilutecoccus | Aerotolerant | - | - |
| Marinimicrobium | Aerotolerant | - | - |
| Marinobacter | Aerotolerant | - | - |
| Marinococcus | Aerotolerant | - | - |
| Maritimimonas | Aerotolerant | - | - |
| Marmoricola | Aerotolerant | - | Correct |
| Martelella | Aerotolerant | - | - |
| Marvinbryantia | Anaerobic | - | Correct |
| Massilia | Aerotolerant | - | Correct |
| Mastigocladopsis PCC-10914 | Aerotolerant | - | - |
| MD3-55 | Unknown | - | - |
| Megamonas | Anaerobic | - | - |
| Meiothermus | Aerotolerant | - | - |
| Mesorhizobium | Aerotolerant | - | Correct |
| metagenome | Unassigned | - | - |
| Methylobacterium | Aerotolerant | - | - |
| Methyloceanibacter | Aerotolerant | - | - |
| Methylocystis | Aerotolerant | - | - |
| Methyloligella | Aerotolerant | - | - |
| Methylophaga | Aerotolerant | - | - |
| Methylopila | Aerotolerant | - | - |
| Methyloversatilis | Aerotolerant | - | - |
| Methylovirgula | Aerotolerant | - | - |
| Microbacterium | Aerotolerant | Correct | Correct |
| Microbulbifer | Aerotolerant | - | - |
| Microlunatus | Aerotolerant | - | Correct |
| Micromonospora | Aerotolerant | Correct | - |
| Micropruina | Aerotolerant | - | - |
| Microvirga | Aerotolerant | - | Correct |
| Minicystis | Aerotolerant | - | - |
| Miniimonas | Aerotolerant | - | - |
| MIZ36 | Unknown | - | - |
| Mizugakiibacter | Aerotolerant | - | - |
| mle1-7 | Unknown | - | - |
| MN 122.2a | Unknown | - | - |
| MND1 | Unknown | - | - |
| Mobilicoccus | Aerotolerant | - | - |
| Mobilitalea | Anaerobic | - | - |
| Modestobacter | Aerotolerant | - | - |
| Moheibacter | Aerotolerant | - | - |
| Moraxella | Aerotolerant | - | Correct |
| Morganella | Aerotolerant | - | Correct |
| Moryella | Anaerobic | - | - |
| Motilibacter | Aerotolerant | - | - |
| Mucilaginibacter | Aerotolerant | - | - |
| Mucispirillum | Anaerobic | Correct | - |
| Mumia | Aerotolerant | - | - |
| Muribaculum | Anaerobic | - | Correct |
| Muricauda | Aerotolerant | - | - |
| Muricoccus | Aerotolerant | - | - |
| Murimonas | Anaerobic | - | - |
| Mycetocola | Aerotolerant | - | - |
| Mycobacterium | Aerotolerant | Correct | Correct |
| Mycoplana | Aerotolerant | - | - |
| Myroides | Aerotolerant | - | Correct |
| Myxococcus | Aerotolerant | - | - |
| Myxosarcina GI1 | Aerotolerant | - | - |
| Myxosarcina SAG 30.84 | Aerotolerant | - | - |
| Nakamurella | Aerotolerant | Correct | Correct |
| Nannocystis | Aerotolerant | - | - |
| Negativicoccus | Anaerobic | - | - |
| Neo-b11 | Unknown | - | - |
| Nesterenkonia | Aerotolerant | - | - |
| Niabella | Aerotolerant | - | - |
| Niastella | Aerotolerant | - | Correct |
| Nitratireductor | Aerotolerant | - | - |
| Nitriliruptor | Aerotolerant | - | - |
| Nitrolancea | Aerotolerant | - | - |
| Nitrosococcus | Aerotolerant | - | - |
| Nitrosomonas | Aerotolerant | - | - |
| Nitrospira | Aerotolerant | - | Correct |
| Nocardia | Aerotolerant | Correct | - |
| Nocardioides | Aerotolerant | - | Correct |
| Nocardiopsis | Aerotolerant | - | - |
| Nonlabens | Aerotolerant | - | - |
| Nonomuraea | Aerotolerant | - | - |
| Nordella | Aerotolerant | - | Correct |
| Nostoc PCC-73102 | Aerotolerant | - | - |
| Noviherbaspirillum | Aerotolerant | - | - |
| Novosphingobium | Aerotolerant | - | Correct |
| Nubsella | Aerotolerant | - | - |
| Oceanicella | Aerotolerant | - | - |
| Oceanimonas | Aerotolerant | - | - |
| Oceanisphaera | Aerotolerant | - | - |
| Oceanobacillus | Aerotolerant | - | - |
| Ochrobactrum | Aerotolerant | - | Correct |
| Odoribacter | Anaerobic | Correct | Correct |
| Ohtaekwangia | Aerotolerant | - | Correct |
| OLB13 | Unknown | - | - |
| OLB15 | Unknown | - | - |
| OLB17 | Unknown | - | - |
| OLB8 | Unknown | - | - |
| Oligella | Aerotolerant | - | - |
| Oligoflexus | Anaerobic | - | Wrong |
| OM27 clade | Unknown | - | - |
| OM60(NOR5) clade | Unknown | - | - |
| Opitutus | Aerotolerant | - | Wrong |
| Orbus | Aerotolerant | - | - |
| Ornithinicoccus | Aerotolerant | - | - |
| Ornithinimicrobium | Aerotolerant | - | - |
| Ornithobacterium | Aerotolerant | - | - |
| Oscillibacter | Anaerobic | - | Correct |
| Oscillochloris | Anaerobic | - | - |
| Oxalobacter | Anaerobic | - | Correct |
| Oxalophagus | Aerotolerant | - | - |
| p-1088-a5 gut group | Anaerobic | - | - |
| Paenalcaligenes | Aerotolerant | - | - |
| Paenibacillus | Aerotolerant | Correct | Correct |
| Paeniclostridium | Anaerobic | Correct | - |
| Paenirhodobacter | Anaerobic | - | - |
| Paenochrobactrum | Aerotolerant | - | - |
| Pajaroellobacter | Aerotolerant | - | - |
| Palleronia | Aerotolerant | - | - |
| Paludibacter | Anaerobic | - | - |
| Paludibaculum | Aerotolerant | - | - |
| Paludisphaera | Aerotolerant | Correct | - |
| Panacagrimonas | Aerotolerant | - | - |
| Pandoraea | Aerotolerant | - | - |
| Pantoea | Aerotolerant | - | Correct |
| Papillibacter | Anaerobic | - | - |
| Parabacteroides | Anaerobic | Correct | Correct |
| Paraburkholderia tropica | Aerotolerant | - | - |
| Paraclostridium | Anaerobic | Correct | - |
| Paracoccus | Aerotolerant | - | - |
| Paraeggerthella | Anaerobic | - | - |
| Parafilimonas | Anaerobic | - | Wrong |
| Parapedobacter | Aerotolerant | - | - |
| Parapusillimonas | Aerotolerant | - | - |
| Pararhodospirillum | Anaerobic | - | - |
| Parasphingopyxis | Aerotolerant | - | - |
| Parasutterella | Aerotolerant | - | Wrong |
| Parviterribacter | Aerotolerant | - | - |
| Pasteuria | Aerotolerant | - | - |
| Patulibacter | Aerotolerant | - | - |
| Pectobacterium | Aerotolerant | - | - |
| Pediococcus | Aerotolerant | - | - |
| Pedobacter | Aerotolerant | - | Correct |
| Pedomicrobium | Aerotolerant | - | Correct |
| Pelagibacterium | Aerotolerant | - | - |
| Pelagibius | Aerotolerant | - | - |
| Peptoclostridium | Anaerobic | - | - |
| Peptococcus | Anaerobic | - | Correct |
| Peptoniphilus | Anaerobic | - | - |
| Peredibacter | Aerotolerant | - | - |
| Persicitalea | Aerotolerant | - | - |
| Phascolarctobacterium | Anaerobic | Correct | - |
| Phaselicystis | Aerotolerant | - | Correct |
| Phenylobacterium | Aerotolerant | - | Correct |
| Phormidium IAM M-71 | Aerotolerant | - | - |
| Photobacterium | Aerotolerant | - | - |
| Phreatobacter | Aerotolerant | - | - |
| Phycicoccus | Aerotolerant | - | - |
| Phyllobacterium | Aerotolerant | - | Correct |
| Phytohabitans | Aerotolerant | - | - |
| Pir3 lineage | Aerotolerant | - | - |
| Pir4 lineage | Aerotolerant | Correct | - |
| Pirellula | Aerotolerant | Correct | Correct |
| Planctomicrobium | Aerotolerant | - | - |
| Planctomycetaceae bacterium Bac131 | Unknown | - | - |
| Planctomycetales bacterium Ellin6207 | Unknown | - | - |
| Planctopirus | Aerotolerant | - | - |
| Planktomarina | Aerotolerant | - | - |
| Pleomorphomonas | Aerotolerant | - | - |
| Pleurocapsa PCC-7319 | Aerotolerant | - | - |
| Pleurocapsa PCC-7327 | Aerotolerant | - | - |
| PMMR1 | Unknown | - | - |
| Polyangium | Aerotolerant | - | - |
| Polymorphobacter | Aerotolerant | - | - |
| Pontibaca | Aerotolerant | - | - |
| Porphyromonas | Anaerobic | - | - |
| Portibacter | Aerotolerant | - | - |
| Pragia | Aerotolerant | - | - |
| Prauserella | Aerotolerant | - | - |
| Prevotellaceae UCG-001 | Anaerobic | - | - |
| Pricia | Aerotolerant | - | - |
| Promicromonospora | Aerotolerant | - | - |
| Propionicicella | Aerotolerant | - | - |
| Propioniciclava | Aerotolerant | - | - |
| Prosthecobacter | Aerotolerant | - | - |
| Proteiniborus | Anaerobic | - | - |
| Proteiniphilum | Anaerobic | - | - |
| Proteus | Aerotolerant | - | - |
| Providencia | Aerotolerant | - | Correct |
| Pseudactinotalea | Aerotolerant | - | - |
| Pseudaminobacter | Aerotolerant | - | Correct |
| Pseudenhygromyxa | Aerotolerant | - | - |
| Pseudoalteromonas | Aerotolerant | - | - |
| Pseudochelatococcus | Aerotolerant | - | - |
| Pseudochrobactrum | Aerotolerant | - | - |
| Pseudoclavibacter | Aerotolerant | - | - |
| Pseudoflavitalea | Aerotolerant | - | - |
| Pseudofulvimonas | Aerotolerant | - | - |
| Pseudogracilibacillus | Aerotolerant | - | - |
| Pseudohoeflea | Aerotolerant | - | - |
| Pseudokineococcus | Aerotolerant | Correct | - |
| Pseudolabrys | Aerotolerant | - | Correct |
| Pseudomonas | Aerotolerant | - | Correct |
| Pseudonocardia | Aerotolerant | - | Correct |
| Pseudopropionibacterium | Aerotolerant | - | - |
| Pseudorhodoplanes | Aerotolerant | - | Correct |
| Pseudovibrio | Aerotolerant | - | - |
| Pseudoxanthobacter | Aerotolerant | - | - |
| Pseudoxanthomonas | Aerotolerant | - | Correct |
| Psychrobacter | Aerotolerant | - | - |
| Psychroglaciecola | Aerotolerant | - | - |
| Psychromonas | Aerotolerant | - | - |
| Pusillimonas | Aerotolerant | - | - |
| Pygmaiobacter | Anaerobic | - | - |
| Pyramidobacter | Unknown | - | - |
| Qipengyuania | Aerotolerant | - | - |
| Quadrisphaera | Aerotolerant | Correct | - |
| Ralstonia | Aerotolerant | - | - |
| Ramlibacter | Aerotolerant | - | - |
| Raoultibacter | Aerotolerant | - | - |
| Rathayibacter | Aerotolerant | - | - |
| RB41 | Unknown | - | - |
| Reyranella | Aerotolerant | - | Correct |
| Rheinheimera | Aerotolerant | - | - |
| Rhizobacter | Aerotolerant | - | Correct |
| Rhizocola | Aerotolerant | - | - |
| Rhizorhapis | Aerotolerant | - | - |
| Rhodanobacter | Aerotolerant | - | Correct |
| Rhodobacter | Aerotolerant | - | - |
| Rhodoblastus | Anaerobic | - | - |
| Rhodococcus | Aerotolerant | Correct | - |
| Rhodocytophaga | Aerotolerant | - | - |
| Rhodoligotrophos | Aerotolerant | - | - |
| Rhodomicrobium | Aerotolerant | - | - |
| Rhodopirellula | Aerotolerant | - | - |
| Rhodoplanes | Aerotolerant | - | Correct |
| Rhodopseudomonas | Aerotolerant | - | - |
| Rhodovarius | Aerotolerant | - | - |
| Rhodovastum | Aerotolerant | - | - |
| Rickettsia | Aerotolerant | - | - |
| Rickettsiella | Aerotolerant | - | - |
| Rikenella | Anaerobic | Wrong | Correct |
| Rikenellaceae RC9 gut group | Anaerobic | - | - |
| Robertkochia | Aerotolerant | - | - |
| Robiginitalea | Aerotolerant | - | - |
| Robinsoniella | Anaerobic | Correct | - |
| Romboutsia | Anaerobic | Correct | Correct |
| Roseburia | Anaerobic | - | Correct |
| Roseiarcus | Anaerobic | - | - |
| Roseibacillus | Aerotolerant | - | - |
| Roseiflexus | Anaerobic | - | - |
| Roseimaritima | Aerotolerant | - | - |
| Roseimicrobium | Aerotolerant | - | - |
| Roseobacter clade CHAB-I-5 lineage | Aerotolerant | - | - |
| Roseomonas | Aerotolerant | - | - |
| Roseovarius | Aerotolerant | - | - |
| Rothia | Aerotolerant | Correct | - |
| Ruania | Aerotolerant | - | - |
| Rubellimicrobium | Aerotolerant | - | - |
| Rubinisphaera | Aerotolerant | - | - |
| Rubripirellula | Aerotolerant | - | - |
| Rubritepida | Aerotolerant | - | - |
| Rubrivirga | Anaerobic | - | - |
| Rubrobacter | Aerotolerant | - | - |
| Rudaea | Aerotolerant | - | - |
| Ruminiclostridium | Anaerobic | - | - |
| Ruminiclostridium 1 | Anaerobic | Correct | - |
| Ruminiclostridium 5 | Anaerobic | Correct | - |
| Ruminiclostridium 9 | Anaerobic | - | - |
| Ruminococcaceae NK4A214 group | Anaerobic | - | - |
| Ruminococcaceae UCG-005 | Anaerobic | - | - |
| Ruminococcaceae UCG-008 | Anaerobic | - | - |
| Ruminococcaceae UCG-009 | Anaerobic | - | - |
| Ruminococcaceae UCG-010 | Anaerobic | - | - |
| Ruminococcaceae UCG-012 | Anaerobic | - | - |
| Ruminococcaceae UCG-013 | Anaerobic | - | - |
| Ruminococcaceae UCG-014 | Anaerobic | - | - |
| Ruminococcaceae V9D2013 group | Anaerobic | - | - |
| Ruminococcus 1 | Anaerobic | - | - |
| Rummeliibacillus | Aerotolerant | - | - |
| Saccharibacillus | Aerotolerant | - | Correct |
| Saccharibacter | Aerotolerant | - | - |
| Saccharopolyspora | Aerotolerant | - | - |
| Saccharothrix | Aerotolerant | - | - |
| Saccharum hybrid cultivar | Unassigned | - | - |
| Salana | Aerotolerant | - | - |
| Salimicrobium | Aerotolerant | - | - |
| Salinicoccus | Aerotolerant | - | - |
| Salinicola | Aerotolerant | - | - |
| Salinisphaera | Aerotolerant | - | - |
| Salipaludibacillus | Aerotolerant | - | - |
| Sandaracinobacter | Aerotolerant | - | - |
| Sandaracinus | Aerotolerant | - | - |
| Sanguibacter | Aerotolerant | - | - |
| Sanguibacteroides | Anaerobic | - | - |
| SAR92 clade | Aerotolerant | - | - |
| Sarcina | Aerotolerant | - | - |
| Savagea | Aerotolerant | - | - |
| SCGC AAA164-E04 | Unknown | - | - |
| Schlesneria | Aerotolerant | - | Correct |
| Sciscionella | Aerotolerant | - | - |
| Scytonema PCC-7110 | Aerotolerant | - | - |
| Scytonema UTEX 2349 | Aerotolerant | - | - |
| Scytonema VB-61278 | Aerotolerant | - | - |
| Sebaldella | Anaerobic | - | - |
| Sedimentibacter | Anaerobic | - | - |
| Sediminibacterium | Aerotolerant | - | - |
| Sediminimonas | Aerotolerant | - | - |
| Sediminivirga | Aerotolerant | - | - |
| Segetibacter | Aerotolerant | - | - |
| Segniliparus | Aerotolerant | - | - |
| Sellimonas | Anaerobic | - | - |
| Serinibacter | Aerotolerant | - | - |
| Serratia | Aerotolerant | - | - |
| SH-PL14 | Unknown | - | - |
| Shewanella | Aerotolerant | - | - |
| Shinella | Aerotolerant | - | - |
| Silvanigrella | Aerotolerant | - | - |
| Singulisphaera | Aerotolerant | Correct | Correct |
| Sinobaca | Aerotolerant | - | - |
| Sinobacterium | Aerotolerant | - | - |
| Sinomicrobium | Aerotolerant | - | - |
| Sinomonas | Aerotolerant | - | - |
| Siphonobacter | Aerotolerant | - | - |
| Skermanella | Aerotolerant | - | - |
| Skermania | Aerotolerant | - | - |
| Slackia | Aerotolerant | - | - |
| SM1A02 | Unknown | - | - |
| Smaragdicoccus | Aerotolerant | - | - |
| Snodgrassella | Unknown | - | - |
| Sodalis | Aerotolerant | - | - |
| soil bacterium WF55 | Unassigned | - | - |
| Solibacillus | Aerotolerant | - | Correct |
| Solirubrobacter | Aerotolerant | - | Correct |
| Solitalea | Aerotolerant | - | Correct |
| Sorangium | Aerotolerant | - | - |
| Sphaerisporangium | Aerotolerant | - | - |
| Sphaerobacter | Aerotolerant | - | - |
| Sphingoaurantiacus | Aerotolerant | - | - |
| Sphingobacterium | Aerotolerant | - | - |
| Sphingobium | Aerotolerant | - | Correct |
| Sphingomonas | Aerotolerant | - | - |
| Sphingopyxis | Aerotolerant | - | - |
| Sphingorhabdus | Aerotolerant | - | - |
| Sphingosinicella | Aerotolerant | - | - |
| Spirochaeta 2 | Anaerobic | - | - |
| Spiroplasma | Aerotolerant | - | - |
| Spirosoma | Aerotolerant | - | - |
| Sporacetigenium | Anaerobic | - | - |
| Sporichthya | Aerotolerant | - | - |
| Sporocytophaga | Aerotolerant | - | Correct |
| Sporomusa | Anaerobic | - | - |
| Sporosarcina | Aerotolerant | - | Correct |
| Stackebrandtia | Aerotolerant | - | - |
| Stanieria PCC-7437 | Aerotolerant | - | - |
| Staphylococcus | Aerotolerant | Correct | Correct |
| Stappia | Aerotolerant | - | - |
| Starkeya | Aerotolerant | - | - |
| Stenotrophobacter | Aerotolerant | - | - |
| Stenotrophomonas | Aerotolerant | - | - |
| Steroidobacter | Aerotolerant | - | Correct |
| Streptococcus | Aerotolerant | - | Correct |
| Streptomyces | Aerotolerant | Correct | Correct |
| Streptosporangium | Aerotolerant | - | - |
| Subdoligranulum | Anaerobic | - | - |
| Subgroup 10 | Unknown | - | - |
| Sulfurovum | Aerotolerant | - | - |
| Sutterella | Anaerobic | - | - |
| Suttonella | Aerotolerant | - | - |
| Sva0081 sediment group | Unknown | - | - |
| Sva0996 marine group | Unknown | - | - |
| Swaminathania | Aerotolerant | - | - |
| SWB02 | Unknown | - | - |
| Symplocastrum CPER-KK1 | Aerotolerant | - | - |
| Synechococcus CC9902 | Aerotolerant | - | - |
| Synechococcus IR11 | Aerotolerant | - | - |
| Synechococcus PCC-7902 | Aerotolerant | - | - |
| Synechocystis BDHKU-20401 | Aerotolerant | - | - |
| Syntrophobacter | Anaerobic | - | - |
| SZB85 | Unknown | - | - |
| Tabrizicola | Aerotolerant | - | - |
| Tagaea | Aerotolerant | - | - |
| Tahibacter | Aerotolerant | - | - |
| Taibaiella | Aerotolerant | - | - |
| Tanticharoenia | Aerotolerant | - | - |
| Telmatocola | Aerotolerant | - | - |
| Tepidamorphus | Aerotolerant | - | - |
| Tepidimonas | Aerotolerant | - | - |
| Tepidisphaera | Aerotolerant | - | - |
| Termite planctomycete cluster | Unknown | - | - |
| Termite Treponema cluster | Anaerobic | - | - |
| Terribacillus | Aerotolerant | - | - |
| Terrimicrobium | Aerotolerant | - | - |
| Terrimonas | Aerotolerant | - | Correct |
| Terrisporobacter | Anaerobic | Correct | - |
| Tessaracoccus | Aerotolerant | - | Correct |
| Tetrasphaera | Aerotolerant | - | - |
| Thauera | Anaerobic | - | - |
| Thermicanus | Aerotolerant | - | - |
| Thermobacillus | Aerotolerant | - | - |
| Thermocatellispora | Aerotolerant | - | - |
| Thermomonas | Aerotolerant | - | - |
| Thermosporothrix | Aerotolerant | - | - |
| Thermus | Aerotolerant | - | - |
| Thiobaca | Aerotolerant | - | - |
| Thioclava | Aerotolerant | - | - |
| Thiodictyon | Aerotolerant | - | - |
| Thiothrix | Aerotolerant | - | - |
| Timonella | Aerotolerant | - | - |
| Tissierella | Anaerobic | - | - |
| TM7 phylum sp. oral clone FR058 | Unknown | - | - |
| Tolypothrix PCC-7601 | Aerotolerant | - | - |
| Tomitella | Aerotolerant | - | - |
| Trabulsiella | Aerotolerant | - | - |
| Tranquillimonas | Aerotolerant | - | - |
| Treponema | Anaerobic | - | Wrong |
| Tropicimonas | Aerotolerant | - | - |
| Truepera | Aerotolerant | - | - |
| Tsukamurella | Aerotolerant | Correct | - |
| Tumebacillus | Aerotolerant | - | - |
| Tunicatimonas | Aerotolerant | - | - |
| Turicibacter | Aerotolerant | - | - |
| Tyzzerella | Anaerobic | Wrong | Correct |
| Tyzzerella 3 | Anaerobic | - | - |
| Tyzzerella 4 | Anaerobic | - | - |
| UBA1819 | Anaerobic | - | - |
| Uliginosibacterium | Aerotolerant | - | - |
| uncultured | Unknown | - | - |
| uncultured Acetobacteraceae bacterium | Aerotolerant | - | - |
| uncultured Acidimicrobidae bacterium | Aerotolerant | - | - |
| uncultured Acidobacteria bacterium | Unknown | - | - |
| uncultured Acidobacteriales bacterium | Unknown | - | - |
| uncultured Acidobacterium sp. | Unknown | - | - |
| uncultured actinobacterium | Unknown | - | - |
| uncultured Actinomycetales bacterium | Unknown | - | - |
| uncultured Alcaligenaceae bacterium | Aerotolerant | - | - |
| uncultured alpha proteobacterium | Unknown | - | - |
| uncultured anaerobic ammonium-oxidizing bacterium | Anaerobic | - | - |
| uncultured Anaerolineaceae bacterium | Anaerobic | - | - |
| uncultured Anaerolineae bacterium | Anaerobic | - | - |
| uncultured Anaeromyxobacter sp. | Aerotolerant | - | - |
| uncultured Archangiaceae bacterium | Aerotolerant | - | - |
| uncultured Armatimonadetes bacterium | Aerotolerant | - | - |
| uncultured bacterium | Unknown | - | - |
| uncultured bacterium 259 | Unknown | - | - |
| uncultured bacterium GC1 | Unknown | - | - |
| uncultured bacterium gp6 | Unknown | - | - |
| uncultured bacterium mle1-12 | Unknown | - | - |
| uncultured bacterium mle1-27 | Unknown | - | - |
| uncultured bacterium SBR1071 | Unknown | - | - |
| uncultured bacterium SBR2013 | Unknown | - | - |
| uncultured bacterium SBR2096 | Unknown | - | - |
| uncultured bacterium SJP-3 | Unknown | - | - |
| uncultured Bacteroidales bacterium | Anaerobic | - | - |
| uncultured Bacteroidetes bacterium | Anaerobic | - | - |
| uncultured beta proteobacterium | Unknown | - | - |
| uncultured Bradyrhizobiaceae bacterium | Unknown | - | - |
| uncultured Caldilineaceae bacterium | Unknown | - | - |
| uncultured Caldilineae bacterium | Unknown | - | - |
| uncultured candidate division SAM bacterium | Unknown | - | - |
| uncultured candidate division SBR1093 bacterium | Unknown | - | - |
| uncultured candidate division SPAM bacterium | Unknown | - | - |
| uncultured candidate division SR1 bacterium | Unknown | - | - |
| uncultured candidate division WS5 bacterium | Unknown | - | - |
| uncultured candidate division WS6 bacterium | Unknown | - | - |
| uncultured Candidatus Saccharibacteria bacterium | Unknown | - | - |
| uncultured Carnobacterium sp. | Aerotolerant | - | - |
| uncultured Chitinivibrionia bacterium | Aerotolerant | - | - |
| uncultured Chloroflexi bacterium | Anaerobic | - | - |
| uncultured Chloroflexus sp. | Anaerobic | - | - |
| uncultured Clostridium sp. | Anaerobic | - | - |
| uncultured compost bacterium | Unknown | - | - |
| uncultured cyanobacterium | Aerotolerant | - | - |
| uncultured Cytophagales bacterium | Aerotolerant | - | - |
| uncultured deep-sea bacterium | Unknown | - | - |
| uncultured Dehalococcoidia bacterium | Anaerobic | - | - |
| uncultured delta proteobacterium | Unknown | - | - |
| uncultured Desulfocaldus sp. | Anaerobic | - | - |
| uncultured Desulfuromonadales bacterium | Anaerobic | - | - |
| uncultured endolithic bacterium | Unknown | - | - |
| uncultured Epsilonproteobacteria bacterium | Aerotolerant | - | - |
| uncultured Erysipelotrichaceae bacterium | Aerotolerant | - | - |
| uncultured Erysipelotrichia bacterium | Aerotolerant | - | - |
| uncultured Firmicutes bacterium | Unknown | - | - |
| uncultured Flavobacteriales bacterium | Aerotolerant | - | - |
| uncultured forest soil bacterium | Unknown | - | - |
| uncultured Frankineae bacterium | Aerotolerant | - | - |
| uncultured gamma proteobacterium | Unknown | - | - |
| uncultured Gemmatimonadetes bacterium | Aerotolerant | - | - |
| uncultured Holophaga sp. | Anaerobic | - | - |
| uncultured Holophagae bacterium | Anaerobic | - | - |
| uncultured Ignavibacterium sp. | Anaerobic | - | - |
| uncultured Litorilinea sp. | Aerotolerant | - | - |
| uncultured marine bacterium | Unknown | - | - |
| uncultured Microgenomates group bacterium | Unknown | - | - |
| uncultured microorganism | Unknown | - | - |
| uncultured Myxococcales bacterium | Aerotolerant | - | - |
| uncultured organism | Unknown | - | - |
| uncultured Phyllobacteriaceae bacterium | Aerotolerant | - | - |
| uncultured Planctomyces sp. | Aerotolerant | - | - |
| uncultured Planctomycetaceae bacterium | Unknown | - | - |
| uncultured Planctomycetales bacterium | Unknown | - | - |
| uncultured planctomycete | Unknown | - | - |
| uncultured prokaryote | Unknown | - | - |
| uncultured proteobacterium | Unknown | - | - |
| uncultured Rhizobiales bacterium | Unknown | - | - |
| uncultured Rhodocyclaceae bacterium | Aerotolerant | - | - |
| uncultured Rhodospirillaceae bacterium | Anaerobic | - | - |
| uncultured Rhodospirillales bacterium | Anaerobic | - | - |
| uncultured Roseobacter sp. | Aerotolerant | - | - |
| uncultured Rubrobacteraceae bacterium | Aerotolerant | - | - |
| uncultured Rubrobacterales bacterium | Aerotolerant | - | - |
| uncultured Rubrobacteria bacterium | Aerotolerant | - | - |
| uncultured rumen bacterium | Unknown | - | - |
| uncultured Saccharibacillus sp. | Aerotolerant | - | - |
| uncultured sludge bacterium | Unknown | - | - |
| uncultured sludge bacterium H5 | Unknown | - | - |
| uncultured sludge bacterium S14 | Unknown | - | - |
| uncultured soil bacterium | Unknown | - | - |
| uncultured Solirubrobacter sp. | Aerotolerant | - | - |
| uncultured Sphaerobacter sp. | Aerotolerant | - | - |
| uncultured Sphingobium sp. | Aerotolerant | - | - |
| uncultured Stella sp. | Unknown | - | - |
| uncultured Syntrophobacteraceae bacterium | Anaerobic | - | - |
| uncultured Syntrophobacterales bacterium | Anaerobic | - | - |
| uncultured Telmatobacter sp. | Anaerobic | - | - |
| uncultured Thermomicrobia bacterium | Unknown | - | - |
| uncultured verrucomicrobium DEV059 | Unknown | - | - |
| uncultured Xanthobacteraceae bacterium | Aerotolerant | - | - |
| unidentified | Unassigned | - | - |
| Urania-1B-19 marine sediment group | Unknown | - | - |
| Uruburuella | Aerotolerant | - | - |
| Vagococcus | Aerotolerant | Correct | - |
| Variibacter | Aerotolerant | - | - |
| Variovorax | Aerotolerant | - | Correct |
| Veillonella | Anaerobic | - | - |
| Verrucomicrobium | Aerotolerant | - | - |
| Verticia | Aerotolerant | - | - |
| Vibrio | Aerotolerant | - | - |
| Vicinamibacter | Aerotolerant | - | Correct |
| Victivallis | Aerotolerant | - | - |
| Virgibacillus | Aerotolerant | - | - |
| Virgisporangium | Aerotolerant | - | - |
| Vitellibacter | Aerotolerant | - | - |
| Vitreoscilla | Aerotolerant | - | Correct |
| Vulcaniibacterium | Aerotolerant | - | - |
| Vulgatibacter | Aerotolerant | - | - |
| wastewater metagenome | Unassigned | - | - |
| Weissella | Aerotolerant | Correct | - |
| Williamsia | Aerotolerant | Correct | - |
| Woeseia | Aerotolerant | - | - |
| Wohlfahrtiimonas | Aerotolerant | - | - |
| Wolbachia | Aerotolerant | - | - |
| Xanthobacter | Aerotolerant | - | - |
| Xanthomonas | Aerotolerant | - | Correct |
| Xenococcus CRM | Aerotolerant | - | - |
| Xenorhabdus | Aerotolerant | - | - |
| Xylella | Aerotolerant | - | - |
| Yangia | Aerotolerant | - | - |
| Zavarzinella | Aerotolerant | - | Correct |
| Zeaxanthinibacter | Aerotolerant | - | - |
| ZOR0006 | Unknown | - | - |
| Zymobacter | Aerotolerant | - | - |
